# Kinetoplastid Phylogenomics Reveals the Evolutionary Innovations Associated with the Origins of Parasitism

**DOI:** 10.1016/j.cub.2015.11.055

**Published:** 2016-01-25

**Authors:** Andrew P. Jackson, Thomas D. Otto, Martin Aslett, Stuart D. Armstrong, Frederic Bringaud, Alexander Schlacht, Catherine Hartley, Mandy Sanders, Jonathan M. Wastling, Joel B. Dacks, Alvaro Acosta-Serrano, Mark C. Field, Michael L. Ginger, Matthew Berriman

**Affiliations:** 1 Department of Infection Biology, Institute of Infection and Global Health, University of Liverpool, Liverpool Science Park Ic2, 146 Brownlow Hill, Liverpool L3 5RF, UK; 2 Pathogen Genomics Group, Wellcome Trust Sanger Institute, Wellcome Trust Genome Campus, Hinxton, Cambridge CB10 1SA, UK; 3 Centre de Résonance Magnétique des Systèmes Biologiques (RMSB), UMR 5536 CNRS, Zone Nord, Bâtiment 4A, Université Bordeaux Segalen, 146, Rue Léo Saignat, 33076 Bordeaux Cedex, France; 4 Department of Cell Biology, University of Alberta, Edmonton, AB T6G 2H7, Canada; 5 Faculty of Natural Sciences, University of Keele, Keele ST5 5BG, UK; 6 NIHR HPRU in Emerging and Zoonotic Infections, Institute of Infection and Global Health, University of Liverpool, 1–5 Brownlow Street, Liverpool L69 3GL, UK; 7 Department of Parasitology, Liverpool School of Tropical Medicine, Pembroke Place, Liverpool L3 5QA, UK; 8 Division of Biological Chemistry and Drug Discovery, College of Life Sciences, University of Dundee, Dow Street, Dundee DD1 5EH, UK; 9 Division of Biomedical and Life Sciences, Lancaster University, Lancaster LA1 4YG, UK

## Abstract

The evolution of parasitism is a recurrent event in the history of life and a core problem in evolutionary biology. Trypanosomatids are important parasites and include the human pathogens *Trypanosoma brucei*, *Trypanosoma cruzi*, and *Leishmania* spp., which in humans cause African trypanosomiasis, Chagas disease, and leishmaniasis, respectively. Genome comparison between trypanosomatids reveals that these parasites have evolved specialized cell-surface protein families, overlaid on a well-conserved cell template. Understanding how these features evolved and which ones are specifically associated with parasitism requires comparison with related non-parasites. We have produced genome sequences for *Bodo saltans*, the closest known non-parasitic relative of trypanosomatids, and a second bodonid, *Trypanoplasma borreli*. Here we show how genomic reduction and innovation contributed to the character of trypanosomatid genomes. We show that gene loss has “streamlined” trypanosomatid genomes, particularly with respect to macromolecular degradation and ion transport, but consistent with a widespread loss of functional redundancy, while adaptive radiations of gene families involved in membrane function provide the principal innovations in trypanosomatid evolution. Gene gain and loss continued during trypanosomatid diversification, resulting in the asymmetric assortment of ancestral characters such as peptidases between *Trypanosoma* and *Leishmania*, genomic differences that were subsequently amplified by lineage-specific innovations after divergence. Finally, we show how species-specific, cell-surface gene families (DGF-1 and PSA) with no apparent structural similarity are independent derivations of a common ancestral form, which we call “bodonin.” This new evidence defines the parasitic innovations of trypanosomatid genomes, revealing how a free-living phagotroph became adapted to exploiting hostile host environments.

## Highlights

• The *Bodo saltans* genome reveals evolutionary changes at the origin of parasitism
• Parasite genomes are streamlined, consistent with a loss of functional redundancy
• Expanded parasite transporter genes reflect a reorientation of membrane function
• Non-homologous, parasite cell-surface proteins evolved from a common ancestor

## Graphical Abstract

**Figure:**
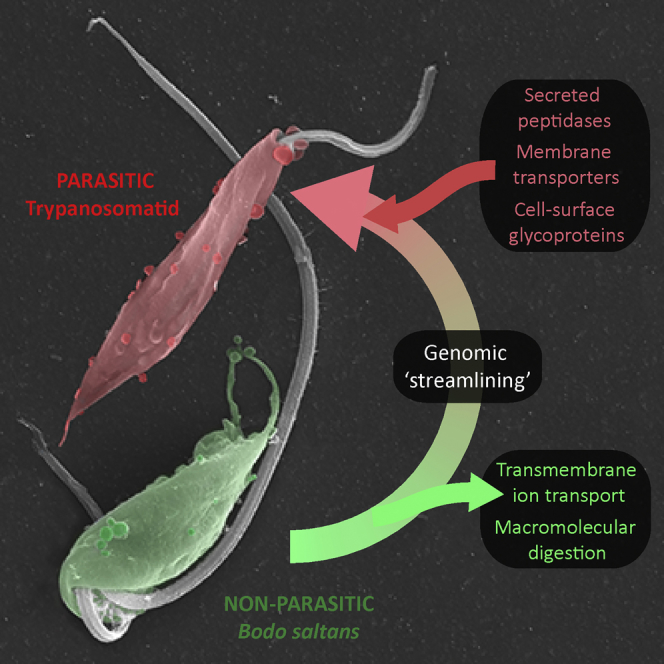

## Teaser

An enduring question in biology is how parasites evolved from free-living organisms. To understand how trypanosomatids became parasitic, Jackson et al. sequenced the genome of a free-living relative (*Bodo saltans*), showing how trypanosomatid genomes became adapted for parasitism through both reduction and elaboration of their free-living legacy.

## Introduction

The history of life is punctuated by the transition from free-living to parasitic organisms, a process often accompanied by profound phenotypic transformation. Parasites are a substantial component of biodiversity, and their origins coincide with major eukaryotic lineages such as Trypanosomatidae, Apicomplexa, Microsporidia, and Neodermata. Parasites affect the fitness of practically every other organism [1], and they have influenced the form and function of all organisms from the earliest times [2].

Phylogenomics provides an opportunity to revisit the engrained view that parasitism is coupled with loss of biological complexity, specialization, and reduced evolutionary capacity [3]. Although celebrated cases such as obligate, intracellular pathogens like *Mycoplasma* [4] and Microsporidia [5] do have much reduced genomes and minimized physiology, most parasite genomes are, in fact, broadly comparable to those of non-parasitic model eukaryotes in size and content. Moreover, all parasite genomes show evidence for innovation and increases in functional complexity. Accurate analysis of the relative contributions of reduction and innovation to parasite evolution requires comparison of parasites not with model organisms, but with closely related non-parasites [6]. Yet, genomes for such free-living relatives are currently uncommon.

Trypanosomatids are a major parasitic lineage with diverse hosts and include the human parasites *Trypanosoma brucei*, *Trypanosoma cruzi*, and *Leishmania* spp. *Bodo saltans* is a free-living Kinetoplastid and the closest known free-living relative of the trypanosomatid parasites [7]. Comparison of the *T. brucei*, *T. cruzi*, and *L*. *major* genome sequences [8, 9, 10] revealed their species-specific features, conspicuous against a background of widespread structural conservation [11, 12]. Many of their shared features could conceivably relate to a parasitic life strategy, such the absence of biosynthetic pathways for haem, purines, and aromatic amino acids. However, without a free-living outgroup for such comparisons, it has been impossible to define shared features that are parasite specific, and therefore plausibly adaptive, and to distinguish these from features shared by kinetoplastids generally.

The absence of a free-living comparator has also impeded an explanation of species-specific features, most obviously the gene families that encode the enigmatic cell-surface proteins specific to each lineage [12], such as the variant surface glycoprotein (VSG) in *T. brucei*, trans-sialidase (TS) and dispersed gene family 1 (DGF-1) in *T. cruzi*, and promastigote surface antigen (PSA) and δ-amastin in *Leishmania* spp. It is unknown whether free-living kinetoplastids possess homologs of these genes and, if so, how they were modified for their prominent role in parasites.

Here we report the *Bodo saltans* genome sequence in comparison with trypanosomatids. We aim to identify the principal genomic changes associated with the ancestral trypanosomatid and so uncover the relative contributions of genomic reduction and innovation to the origin of parasitism. We show that although trypanosomatid genomes have become “streamlined,” gene families crucial for nutrient scavenging and host interactions have been elaborated, and we demonstrate that the enigmatic cell-surface gene families of different parasites originated through radical reorganization of common ancestral structures.

## Results

### Comparative Analysis of Kinetoplastid Genomes

The 39.9 Mb genome of *Bodo saltans* strain Konstanz was sequenced to 170× coverage using Illumina HiSeq. The genome was assembled into 2,402 scaffolds (N50 = 31.5 kb) and includes 18,936 predicted protein-coding sequences. To prevent contamination of the assembly from xenic culture, we restricted the *B. saltans* genome to contigs that contain both eukaryotic homologs and transcriptomic coverage (see the Supplemental Experimental Procedures). The genome sequences of *B. saltans* and model trypanosomatids are compared in Table 1; based on CEGMA (core eukaryotic genes mapping approach) score [13], the *B. saltans* sequence displays a comparable degree of completeness to the reference genomes.

The parasite sequences are 18%–34% shorter than the *B. saltans* genome (Table 1), but they have 41%–56% fewer genes dependent on species, indicating that the parasite genomes are less gene dense. In fact, *B. saltans* has roughly twice as many genes as a parasite but packs them into a comparable space, due to the expansion of non-coding DNA in the parasites. Relative to *B. saltans*, intergenic regions are 63.7%, 55.0%, and 77.3% longer in *T. brucei*, *T. cruzi*, and *L. major*, respectively.

**Figure 1:**
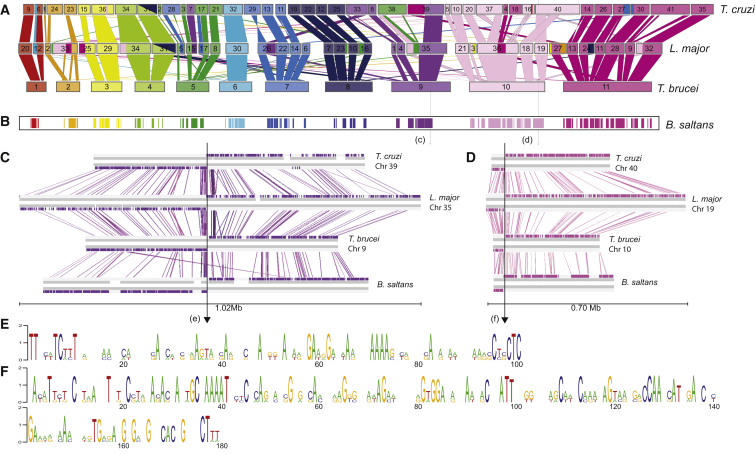
Structural Conservation among Kinetoplastid Genomes and a Putative, Ultraconserved Transcriptional Promoter (A) A cartoon depicting chromosomes of the *T. cruzi* Non-Esmeraldo, *L. major* Friedlin, and *T. brucei* 927 genomes as rectangles, according to scale. *T. brucei* chromosomes are color coded and in ascending order. *T. cruzi* and *L. major* chromosomes are ordered to maximize co-linearity of homologous sequences between species. Homologous sequences are linked by vertical columns that are similarly shaded. (B) Regions of the *B. saltans* and *T. brucei* genome sequences that display co-linearity. Co-linear regions are marked and color coded by the *T. brucei* chromosome to which they correspond. Black arrows indicate two strand-switch regions (SSRs) in the trypanosomatid genomes that are conserved in *B. saltans*. (C and D) Conserved SSRs compared across four genomes. DNA strands are shown as horizontal gray lines adjacent to the coding sequences. The *B. saltans* sequence represents a scaffold of contigs separated by sequence gaps, which are indicated, but physically linked by read pairs. Homologous coding sequences are linked by vertical colored lines. The strand switches are indicated by black arrows. We identified a 102 bp region (E) and a 180 bp region (F) that display 45% and 42% sequence identity, respectively, across the four genomes. (E and F) Consensus nucleotide sequence generated using Weblogo [15] at the SSRs in (C) and (D). See also Data S1.

Transcription in trypanosomatids is polycistronic, and the genome is organized into conserved polycistronic transcription units (PTUs) [11]. To compare genome structure between trypanosomatids and *B. saltans*, we separated *B. saltans* contigs according to sequence similarity with *T. brucei* 927 chromosomes and then aligned each *T. brucei* chromosome and its corresponding *B. saltans* contig bin using wgVISTA [14]. The widespread conservation of genomic position among trypanosomatids [11] does not extend to *B. saltans*; only 1,743 (9.2%) *B. saltans* genes display co-linearity with trypanosomatids, and these are arranged in 157 regions with an average size of 45,240 bp (Figures 1A and 1B; Data S1, sheet 1).

Although generally exceptional, these rare co-linear regions permit us to address the conservation of genome regulation through comparison of non-coding DNA. We examined the two largest co-linear regions (Figures 1C and 1D), which both contain a conserved strand-switch region (SSR) occurring at the junction between PTUs. Within the intergenic region between the PTUs in *B. saltans* and the trypanosomatids, we identified two GA-rich regions of 102 bp (Figure 1E) and 180 bp (Figure 1F), respectively, that display 45% and 42% sequence identity respectively across the four genomes. Polypurine tracts and especially poly(dA:dT) are features of eukaryotic proximal promoter regions [16]. Given that divergent SSRs in *T. brucei* are also known to contain GA-rich transcription initiation sites [17], we suggest that these two regions represent exceptionally well-conserved regulators of transcription common to free-living and parasitic kinetoplastids. This is further supported by the presence, in the second consensus (Figure 1F), of a CAAAT-like motif, which in yeast is bound by transcription factors and is frequently associated with bidirectional promoters [18].

### Gene Clustering Analysis of Genomic Repertoire

Since *B. saltans* has roughly twice the number of parasite genes, this might suggest gene loss in the parasites. To determine all gene losses or gains, we used OrthoMCL [19] to sort *T. brucei*, *T. cruzi*, *L. major*, and *B. saltans* gene products into homologous clusters. We also sequenced the genome of *Trypanoplasma borreli*, a parabodonid parasite of fish. *B. saltans* is a closer relative of trypanosomatids than is *T. borreli* [7]; therefore, any genes shared by both *B. saltans* and *T. borreli* but absent from trypanosomatids will most likely represent a gene loss in the latter, rather than a *B. saltans*-specific gene gain. The *T. borreli* genome was sequenced to draft standard using Illumina HiSeq; the 25.8 Mb genome assembly includes 23,265 contigs (mean contig size = 1,109 bp and N50 = 12,100 bp).

**Figure 2:**
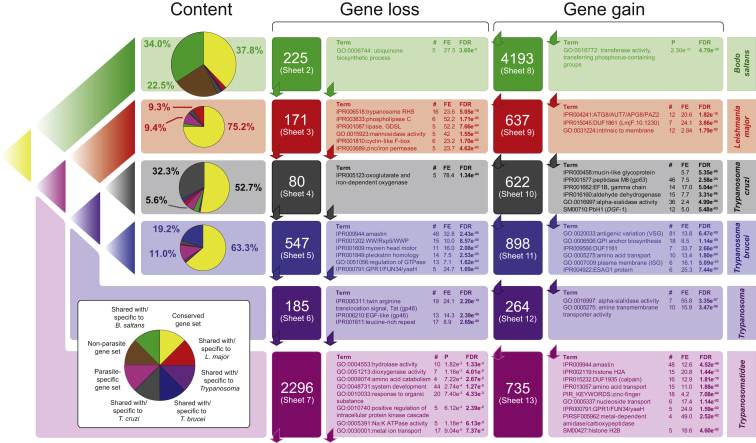
Cluster Analysis of Gene Repertoires, in the Context of Kinetoplastid Phylogeny The phylogeny of four kinetoplastids is depicted top left. Six clades are derived from this and are color coded: *B. saltans* (green), *L. major* (red), *T. cruzi* (black), *T. brucei* (blue), trypanosomes (purple), and trypanosomatids (i.e., all parasites; pink). Immediately to the right are pie charts describing the phylogenetic distribution of gene clusters from the OrthoMCL [19] analysis for each species. Yellow shading denotes clusters that are universally conserved. Brown shading denotes gene clusters found in *B. saltans* and other eukaryotes, but not trypanosomatids (i.e., the non-parasite gene set). Other shading denotes clusters that are species specific or shared with a specific species; e.g., the green segment in the *B. saltans* pie chart denotes *B. saltans*-specific clusters, and green shading elsewhere denotes clusters shared with *B. saltans* only. Gene clusters that have been lost and gained by each clade are indicated further to the right. The supplemental information in Data S1 listing the clusters concerned is noted in each case. Each number is accompanied by the results of enrichment tests on those gene clusters. These report structural and functional terms over-represented among the genes concerned, recording the number involved (#), the fold enrichment (FE), and the p value corrected for the false discovery rate (FDR). See also Figure S1 and Data S1.

The results of clustering analysis are summarized in Figure 2, and the clusters gained and lost by each clade are described in Data S1. We identified a conserved gene set present in *B. saltans* and at least one trypanosomatid, which included 37.8% of *B. saltans* genes and 52.7%–75.2% of all parasite genes. These most likely represent ancestral kinetoplastid genes that were retained after the origin of parasitism. Conversely, a “parasite-only” gene set, present in all trypanosomatids, but not in *B. saltans*, *T. borreli*, or any other eukaryote, included 5.6%–11.0% of all parasite genes. These represent innovations that arose in the last common trypanosomatid ancestor and so are most likely to be associated with the origin of parasitism. Finally, a “non-parasite” gene set present in *B. saltans* and *T. borreli* (or another eukaryotic genome) but absent from all trypanosomatids comprised 4,256 genes or 22.5% of *B. saltans* genes. These genes represent unambiguous gene losses in trypanosomatids after the origin of parasitism.

### Gene Loss: Streamlining of Trypanosomatid Gene Repertoires

If the evolution of parasitism results in obsolescence and loss of functions, then such genes are most likely to be among the “non-parasite” genes. Semantic clustering of Gene Ontology (GO) terms belonging to these (Figure S1) suggests a tendency toward transferase and hydrolase functions and membrane transport. GO terms that are significantly enriched among “non-parasite” genes (Figure 2, pink shading under “gene loss”) include “hydrolase activity” (n = 10; p = 0.001), “aromatic amino acid catabolism” (n = 4; p = 0.027), and “response to organic substance” (n = 20; p = 0.004) (primarily concerning lipid catabolism), as well as “metal ion transport” (GO: 0030001; n = 17; p = 7.37e-4) and “Na:K-exchanging ATPase activity” (GO: 0005391; n = 5; p = 0.006), due to abundant voltage-gated ion channels.

Another approach to identifying gene loss is to compare how all *B. saltans* and trypanosomatid genes map to Kyoto Encyclopedia of Genes and Genomes (KEGG) pathways (Table S1). We find that *B. saltans* does not possess any additional KEGG pathways. In three cases, the parasites have lost genes, resulting in a smaller pathway and minor functional changes: (1) “β-alanine metabolism,” where loss of β-ureidopropionase (Bsal_92075c), dihydropyrimidinase (Bsal_12820), and dihydropyrimidine dehydrogenase (Bsal_90400c) precludes conversion of β-alanine into uracil; (2) “tyrosine metabolism,” where loss of 4-hydroxyphenylpyruvate dioxygenase (Bsal_33500), homogentisate 1,2-dioxygenase (Bsal_09310), 4-Maleylacetoacetate isomerase (Bsal_85365), and two fumarylacetoacetases (Bsal_80270/81380) precludes conversion of tyrosine into fumarate; and (3) “N*-*glycans biosynthesis,” where loss of three alpha-glucosyl-transferases (*Alg6* [Bsal_69160], *Alg8* [Bsal_80175], and *Alg10* [Bsal_31030]) (Figure S2) and the enzyme that makes the donor molecule glucosylphosphoryldolichol (Glcβ-P-Dol) (*Alg5* [Bsal_22320]) indicates that *B. saltans*, like most eukaryotes but unlike trypanosomatids [20], can add alpha-glucose to proteins.

**Figure 3:**
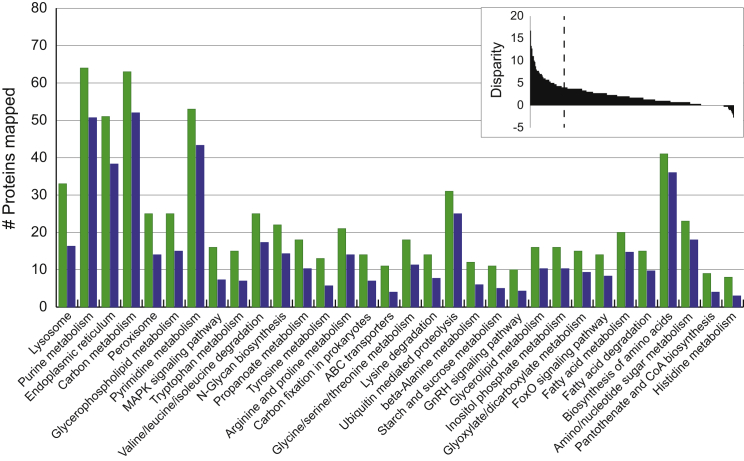
Comparative Mapping *B. saltans* and *T. brucei* Protein Sequences to KEGG Pathways The number of *T. brucei* proteins mapping to individual KEGG terms was subtracted from the corresponding number of *B. saltans* proteins. The disparities for individual KEGG terms are shown in decreasing order (inset). Most KEGG terms have an excess of *B. saltans* proteins mapped. A histogram showing the identities of the top 10% most disparate KEGG terms (to the left of the dashed line, inset) is shown with *B. saltans* gene numbers in green and *T. brucei* gene numbers in blue. See also Table S1 and Figure S2.

Although *B. saltans* cannot apparently perform substantially more physiological functions than trypanosomatids, it possesses a greater number of components in almost all pathways that they share. The greatest disparities in component number concern macromolecular degradation (lysosome/peroxisome) and the catabolism of various metabolites (Figure 3). Among those genes absent from trypanosomatids are diverse proteases and glucosidases normally associated with the lysosome, lysosomal acid lipase (Bsal_14640), and the lysosomal membrane protein, cystinosin (Bsal_81590). Enzymes such as β-glucosidases, α-trehalase (Bsal_69605), and glucoamylases (Bsal_25150 and Bsal_65665) that are absent in the parasites probably allow *B. saltans* to degrade the diverse polysaccharides in bacterial cell walls. Hence, the principal differences in gene content appear to reflect the need for *B. saltans* to degrade bacterial prey within feeding vacuoles and assimilate the products.

Together, these results suggest that there has been a consistent reduction in complexity of numerous pathways in trypanosomatids, most obviously in catabolism, macromolecular degradation, and ion transport, but that the evolution of parasitism did not lead to the widespread loss of metabolic pathways in trypanosomatids. Hence, elements of eukaryotic physiology that are absent in trypanosomatids, such as purine biosynthesis and a glutathione-based system of redox homeostasis, represent ancestral features of kinetoplastids, rather than genetic losses in trypanosomatids. The relatively simple repertoires supporting canonical eukaryotic pathways in trypanosomatids, e.g., SNARE proteins in intracellular trafficking (Figure S3), are no more elaborate in *B. saltans.* Therefore, this simplicity reflects diversity in eukaryotic physiology, rather than genetic losses associated with parasitism; a similar conclusion emerged from comparison of parasitic Apicomplexa and free-living Chrompodellids [21].

### The Phylodiversity of Conserved Gene Families Has Declined in Parasite Genomes

**Figure 4:**
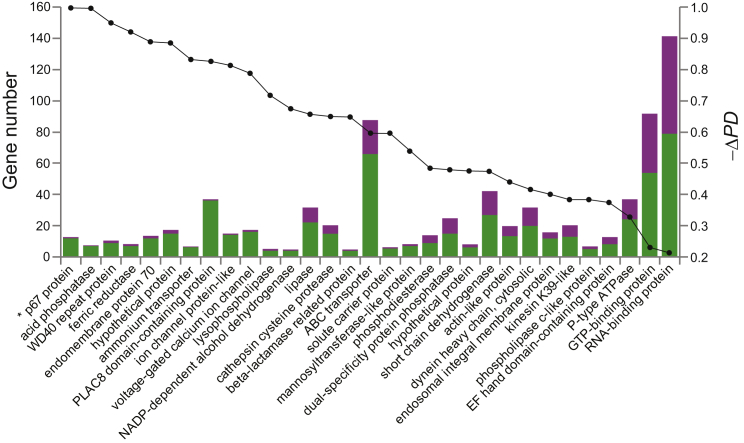
Loss of Phylodiversity in Trypanosomatid Gene Families Relative to *B. saltans* Cluster analysis showed that selected, conserved gene family cases displayed substantial disparities in family size when compared between *B. saltans* and any trypanosomatid. These cases are shown on the x axis, ordered according to the observed loss of phylodiversity in the parasites. On the left, the number of genes in a given family is plotted; *B. saltans* component is shaded green, and the average gene number across three trypanosomatids is shown in pink. On the right, the change in phylodiversity (−ΔPD; see the Supplemental Experimental Procedures), which was uniformly negative, is shown on a scale between 0 and 1, with a value of 1.0 meaning that 100% of diversity was lost in trypanosomatids relative to *B. saltans*. In all cases, the additional *B. saltans* genes were shown to have homologs in other non-kinetoplastids, confirming that they represent parasite losses, rather than *B. saltans*-specific gains. See also Table S2 and Figures S3 and S4.

If trypanosomatids genomes are “streamlined,” the phylogenetic diversity of widely conserved gene families should be reduced. We tested this prediction by estimating phylogenies for clusters in the conserved gene set and comparing their phylogenetic diversity (PD) [22] in trypanosomatids and *B. saltans*. The phylogenetic diversity of *B. saltans* gene families was significantly greater than among their trypanosomatid homologs (Figure 4; Table S2; p = 0.018); not only are *B. saltans* gene families larger, but also they include more diverse evolutionary lineages. Among the largest reductions in PD were gene families associated with hydrolysis (e.g., cathepsin cysteine proteases [reduced by 67%] and lipases [reduced by 66%]), as well as ion transport (e.g., voltage-gated ion channels, reduced by 78% from ten lineages in *B. saltans* to one lineage in *T. brucei*) and membrane transport (e.g., ATP-binding cassette [ABC] transporters, reduced by 60% from 36 lineages in *B. saltans* to 22 lineages in *T. brucei*).

There are also examples of gene losses occurring independently in *Trypanosoma* and *Leishmania*, indicating that genomic reduction continued during trypanosomatid diversification, often resulting in the asymmetric assortment of ancestral gene repertoires among the parasite lineages. For instance, *Trypanosoma* and *Leishmania* each possess two lineages of cathepsin (B and L). When these are compared with *B. saltans* cathepsins (Figure S4), cathepsin-L from *Trypanosoma* and *Leishmania* are orthologous but cathepsin-B is drawn from distinct cysteine peptidase lineages. Asymmetric assortment of the ancestral repertoire most likely affects diverse multi-copy gene families, and it is evident among the secreted peptidases and cell-surface glycoproteins described below. Interestingly, transposable elements have also been retained asymmetrically. The *B. saltans* genome contains all of the previously identified transposable elements in trypanosomatids, namely *ingi* (retroposon, found in all species), SLACS/CZAR (site-specific retroposon of CRE clade, found in most species), VIPER (YR retrotransposon, found in *Trypanosoma* only), and TATE (found in *Leishmania* only). Furthermore, phylogenetic analyses of the bodonid sequences (data not shown) demonstrate that VIPER and TATE belong to a common lineage. Hence, in several respects, *Trypanosoma* spp. and *Leishmania* spp. genomes are independent samples of a larger, ancestral gene repertoire.

### Gene Gain: The Origins of Parasite Adaptations

The abundance of gene clusters that are unique to one or more parasites (Figure 2) shows that the evolution of parasitism involved more than gene loss; gene gain must have a significant role in explaining parasitic origins. Species-specific clusters are dominated by cell-surface-expressed genes and reaffirm the view that the divergence of trypanosomatid genomes was dominated by the rapid evolution of multi-copy gene families [11, 12]. Thus, *L. major*-specific genes are enriched for amastin and PSA (Data S1, sheet 5), whereas *T. cruzi*-specific genes are enriched for mucins, trans-sialidase, GP63, and elongation factor 1β (Data S1, sheet 7). Besides these, the novelty of the *B. saltans* genome is that it allows us to identify “parasite-only” genes (Data S1, sheet 13), i.e., innovations shared by all trypanosomatids that evolved early in their common ancestor. Naturally, as we sample more widely and discover orthologs to these genes in other non-parasitic kinetoplastids, this cohort might be reduced. Nonetheless, these 714 gene clusters are enriched for functional terms associated with membrane transport, primarily of amino acids and nucleic acids, the transmembrane glycoprotein amastin, calpain cysteine peptidases, and nucleosomes (due to various multi-copy, parasite-specific histones).

**Figure 5:**
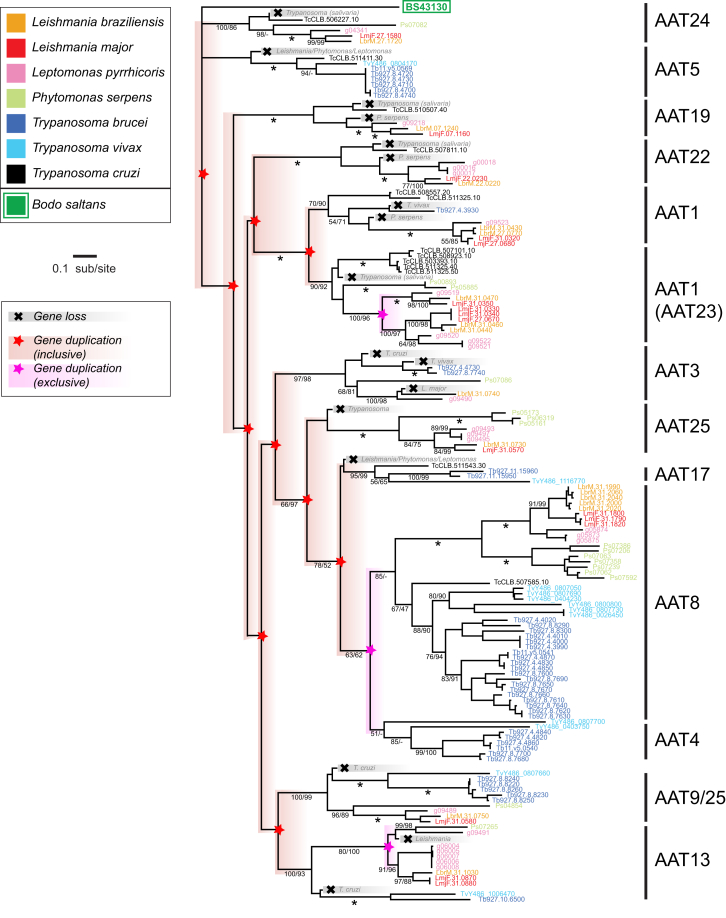
Maximum-Likelihood Phylogeny of Amino Acid Transporter Genes in Kinetoplastids, Estimated from Amino Acid Sequences using a LG + Γ Model Terminal nodes are labeled with gene identifiers and shaded according to species. Clades are labeled on the right according to conserved amino acid transporter loci identified previously [23]. Gene duplications and losses are inferred following reconciliation with a species phylogeny. Black stars indicate putative gene losses, assuming complete sampling. Red stars indicate a putative gene duplication event that includes all species, except where lost subsequently (i.e., which occurred in the common trypanosomatid ancestor). Pink stars indicate a duplication event that occurred after diversification of trypanosomatids and so involves only a subset of species. Duplications affecting only single species are not shown. Bootstrap values for maximum-likelihood (left) and neighbor-joining (right) analyses are shown below subtending branches; for clarity, terminal node support is not shown, although this was uniformly reliable. The tree is rooted using the clade containing the single *B. saltans* homolog. See also Figure S5.

The enrichment analysis points to several dramatic gene family expansions that collectively represent the seminal developments during the period of nascent parasitism. Phylogenetic analysis of amino acid transporter genes suggested previously that these had experienced substantial innovation in the past [23]. By including *B. saltans* genes in the amino acid transporter phylogeny (Figure 5) and reconciling this with the species tree using NOTUNG [24], we now predict that 14 loci were created through gene duplication in the genome of the ancestral trypanosomatid from just a single ancestral locus, prior to the diversification of extant genera. A similar pattern is seen among nucleoside transporters, of which four loci are predicted by phylogenetic reconciliation to have been in the ancestral parasite (Figure S5A), and among amastin glycoproteins. An expansion in δ-amastin has previously been implicated in the evolution of vertebrate parasitism by *Leishmania* [25]. *B. saltans* has multiple copies of amastin that are monophyletic but fall outside of all trypanosomatid sequences in a phylogeny (Figure S5B). This suggests that expansion of δ-amastin in *Leishmania* was preceded by an earlier differentiation of the α, β, γ, and proto-δ-amastin sub-families in the last common ancestor of trypanosomatids, further implicating this poorly understood family in host-parasite interactions.

Peptidases are potent parasite effectors, crucial to initiating infection and evading immunity. Despite the loss of ancestral cathepsins (see above), all parasites have subsequently duplicated the remaining cathepsin-L gene. Calpain cysteine peptidases, although present in *B. saltans*, have also experienced several independent expansions in trypanosomatids, such that they are enriched among “parasite-only” genes (Figure 2; Data S1, sheet 13). Finally, the major surface protease (MSP or gp63) gene family has been substantially modified after the origin of parasitism. MSPs perform multiple roles in maintaining infection and ensuring transmission [26]. There are several *MSP* loci in *B. saltans* that, like the diverse calpains and cathepsins, probably have important proteolytic roles inside digestive vacuoles. MSP phylogeny (Figure S5C) demonstrates that both *Trypanosoma* and *Leishmania* have independently expanded their MSP repertoires relative to *B. saltans*.

To summarize, these various innovations represent early changes to the ancestral trypanosomatid coincident with, or closely following, the transition to parasitism. These predate the origins of enigmatic multi-copy gene families such as VSG, PSA, and DGF-1 that now characterize the genomes of distinct trypanosomatid lineages. Unlike the innovations described thus far, these species-specific genes families are entirely novel, with unknown origins and no obvious structural affinities until now.

### Bodonin: A Multi-copy Family of Transmembrane Glycoproteins

*B. saltans* possesses a multi-copy gene family that we call “bodonin.” The largest bodonin genes encode predicted glycoproteins with a transmembrane domain (TMD) averaging seven transmembrane helices, preceded by a predicted extracellular domain (ED) and followed by a predicted intracellular domain (ID) (Figure S6). We refer to this complete topology as the canonical form (n = 394). There are ∼1,100 additional bodonin genes encoding the ED only, although sequence gaps make the accuracy of these gene models uncertain. The ED is repetitive, with abundant Ser, Thr, and Asn residues (on average, 14.8%, 10.4%, and 5.6% by content); glycosylation of these residues is predicted to produce highly processed glycoproteins.

**Figure 6:**
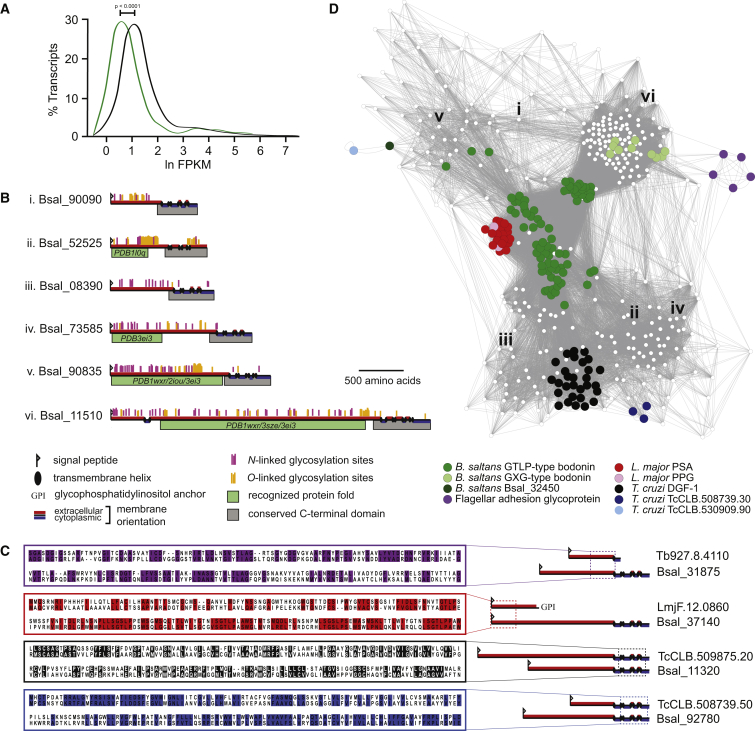
Diversity, Structure, and Expression of the Bodonin Gene Family (A) Transcript abundance of canonical bodonin genes extracted from a whole-genome transcriptomics analysis using Cufflinks. The frequency distribution for bodonin (green line) is compared for all other *B. saltans* genes (black line). (B) Proteomic analysis identified six full-length bodonin isoforms. These are listed alongside their gene identifiers and cartoons of their predicted protein structures, shown to scale. Predictions for signal peptides, transmembrane domains, membrane orientation, and glycosylation sites are shown (see the Supplemental Experimental Procedures). Also shown (in green) are recognized protein folds (with their Protein Data Bank identifiers) with which these bodonin proteins display significant similarity, as determined using pGenThreader (see the Supplemental Experimental Procedures). (C) Sequence conservation between FLA1 (purple), PSA (red), DGF-1 (black), and a *T. cruzi* hypothetical protein (blue) and their closest bodonin homologs, relating to defined regions shown in cartoon form at right. Identical and similar residues are shaded. (D) A Cytoscape network of canonical bodonin genes (n = 394) based on similarity scores generated using HMMER [27]. Sub-families of note are color coded; other bodonin genes are depicted in white. The positions of six expressed isoforms (in B) are indicated with Roman numerals. See also Figure S6.

Transcriptomic analysis suggests that all bodonin genes are constitutively transcribed. However, bodonin transcript abundance has a mean average significantly lower than that of all transcripts (Figure 6A), suggesting that most bodonin proteins have relatively low abundance. Despite enriching whole-cell fractions for glycoproteins and for transmembrane proteins, we observed only ten bodonin proteins in proteomic analyses, and these were represented by only one or two peptides (Figure 6B). Given that hundreds of bodonin genes are transcribed, we suggest that many more bodonin proteins were expressed, but below the level of detection sensitivity. Analysis of protein folding predicts significant similarity between ED tertiary structures and bacterial autotransporter or beta barrel-like domains, known to possess adhesin properties (Figure 6A; see the Supplemental Experimental Procedures).

Bodonin proteins have other structural similarities (Figure 6C). The TMDs of some bodonins resemble DGF-1 proteins, a lineage-specific multi-copy family from *T. cruzi* [28]. Meanwhile, the EDs of other bodonin copies are related to the *Leishmania*-specific PSA protein family [29]. DGF-1 has a similar structure to canonical bodonin, with a large, glycosylated ED, TMD, and ID [28]. PSA, however, is attached to the plasma membrane with a glycosylphosphatidylinisotol (GPI) anchor and has no TMD or ID [29] (Figure S6). Thus, although DGF-1 and PSA lack obvious homology, they do display similarity with distinct domains of the canonical bodonin structure. This suggests a complex evolutionary history, with DGF-1 and PSA having arisen through independent derivations of a bodonin cell-surface protein in the ancestral trypanosomatid.

We used a bodonin hidden Markov model (HMM) to search trypanosomatid genomes for further bodonin-like genes and created a network from HMM scores (Figure 6C). The network confirms that DGF-1 and PSA are related to different parts of the bodonin repertoire, and it reveals other bodonin-like proteins in *T. cruzi* yet to be characterized (e.g., TcCLB.530909.90). Another class of bodonin ED (“GXG”) is homologous to the flagellar adhesion glycoprotein FLA1 in *T. brucei* (GP72 in *T. cruzi*), which is a component of the flagellar attachment zone (FAZ) [30]. Altogether, there are at least five independent derivations from bodonin in trypanosomatids, which provide an evolutionary link between diverse glycoproteins that, after radical transformation, no longer have obvious homology.

## Discussion

The *B. saltans* genome sequence overcomes an historic limitation in understanding how trypanosomatid parasites evolved, allowing us to differentiate events that occurred in the common trypanosomatid ancestor, roughly coincident with the origin of parasitism, from the many important developments that occurred subsequently in distinct parasite lineages. Our analysis reconstructs the genome of the ancestral trypanosomatid, which might ultimately be illuminated by the recent description of the most basal-branching trypanosomatid, *Paratrypanosoma confusum* [31]. However, as an obligate parasite of insects (much like more derived genera, e.g., *Crithidia*), *P. confusum* does not display the facultative parasitic or commensal habit we might reasonably expect of a nascent parasite. Nevertheless, the habits of diverse bodonids suggest that the ancestral trypanosomatid evolved from a phagotroph employing holozoic nutrition [32]. Having abandoned this, it appears to have lost genes that functioned in macromolecular digestion and assimilation, as well as intracellular membrane pumps and ABC transporters, which we suggest functioned previously to transport nutrients and waste across vacuolar membranes. Coupled with the multiplication of membrane transporters for scavenging amino acids, nucleosides, and other metabolites from the host, these changes perhaps reflect a major reorientation of membrane function in the parasites from transport from within (i.e., across the vacuolar membrane) to transport from without (i.e., across the plasma membrane).

However, minimization of physiology and widespread loss of metabolism did not occur, we suggest largely because the heterotrophic, free-living ancestor itself lacked most biosynthetic capabilities. Rather, the genome has been “streamlined,” which we interpret as evidence for loss of functional redundancy. Studies in yeast have indicated that functional redundancy results from gene duplications that are retained under purifying selection to provide “backups” to protect against environmental perturbation [33]. In the absence of such perturbation, both mutualistic and pathogenic endocytic bacteria lose redundancy through a contraction of gene families, while the physiological capacity is often maintained [34]. We suggest that functional redundancy was also lost in the ancestral trypanosomatid as it moved from an abiotic to a biotic environment, with a narrower physiological range and fewer environmental perturbations.

The genomes of some eukaryotic parasites appear to fulfil the expectation of genome reduction when compared with model organisms. Thus, some Microsporidian genomes (though not all) are described as being reduced to a physiological minimum [5]. Other examples might include the morphologically reduced cestodes, which lack homeobox gene families associated with animal development [35]. However, it is possible that genomic reduction in both cases had already begun prior to obligate parasitism [35, 36]. In fact, when parasites are compared with free-living relatives, the differences have been less dramatic. Comparison of apicomplexan parasites with free-living chromerids and colpodellids has shown that this origin of parasitism coincided with some metabolic losses such as de novo purine and tryptophan biosynthesis, but that photosynthesis was abandoned long before [21]. Much like the Kinetoplastida, asymmetric and lineage-specific losses continued during apicomplexan diversification [37], but there is little to distinguish the physiology of the ancestral apicomplexan from its chrompodellid sister taxa [21, 37], while parasite innovations are focused primarily on cell-surface features or secretory products [21]. Analyses of the parasitic ciliate *Ichthyophthirius multifiliis* [38] and the non-photosynthetic alga *Helicosporidium* [39] showed that neither parasite is dramatically reduced relative to its free-living comparators, but that gene family diversity has declined in both, similar to the effect seen here. Essentially, the evidence for genomic reduction through physiological minimization is idiosyncratic; what is lost varies case by case. However, evidence from diverse taxa suggests that genomic streamlining may be a more ubiquitous response to the evolution of obligate parasitism or, indeed, mutualism.

Like reduction, specialization has long been considered a defining feature of parasites, and the diverse, lineage-specific proteins found on trypanosomatid cell surfaces are a pertinent example [11, 12]. Among bodonin genes in *B. saltans* are homologs of PSA in *Leishmania* spp., DGF-1 in sterorarian *Trypanosoma*, and FLA1 across the Trypanosomatidae. These proteins have distinct structures and roles. DGF-1 genes encode abundant transmembrane glycoproteins with conserved integrin motifs, suggestive of a role in cell adhesion [28]. FLA1 is essential for attachment of the flagellum to the *T. brucei* cell body [30]. PSA genes encode highly immunogenic leucine-rich repeat proteins that probably bind other proteins, e.g., host complement [40]. The related proteophosphoglycan (PPG) gene family encodes mucin-like glycoproteins implicated in establishment in the insect vector after transmission [41]. Despite their diverse roles, adhesion and binding properties are a common theme running through all these derivatives and, indeed, bodonin itself (Figure 6B). We speculate that diverse bodonins cover the *B. saltans* cell surface, allowing attachment to both prey and substrata during feeding. For the first time, we have identified the origins of enigmatic parasite gene families in a non-parasitic relative and shown how apparently non-homologous proteins can evolve from a common ancestral form, in this case modifying an adhesin required perhaps to capture prey, to instead bind host cells and proteins.

Bodonin represents a precursor of the prolific, multi-copy gene families so characteristic of trypanosomatid genomes. Thus, the evolution of such families does not define the origin of parasitism per se. However, there are clear differences in how the gene families are organized and in the qualities of the surface coats they most likely produce. There is no evidence yet that bodonin is a contingency gene family, with sophisticated mechanisms for developmental regulation. Indeed, such regulation in trypanosomatids may be the seminal parasitic innovation rather than the genes themselves.

### Conclusions

This study explains how a free-living phagotroph inhabiting diverse and labile surroundings could have become an obligate parasite exploiting a series of relatively constant host environments. There were no major losses in function, but there was streamlining of functional redundancy as the physiological range inhabited by the organism became narrower. The ancestral trypanosomatid also elaborated gene families crucial in scavenging micronutrients and host invasion, part of a radical specialization of the cell surface to meet the demands of transmission and host interaction. This emphasizes the essential feature of becoming parasitic: the environment becomes responsive. Hence, the dominant factor in trypanosomatid evolution became the host immune system, unleashing a selective pressure that constantly challenges the parasite surface to this day. This interaction provides a compelling record of coevolution, a testament to the ability of hosts to shape parasite biology and of parasites to survive those assaults.

## Experimental Procedures

### Genome Strains and Cell Culture

*Bodo saltans* Konstans was isolated from Lake Konstanz, Germany in 2007 and kindly donated by Professor Julius Lûkes (University of South Bohemia). Subsequently, it was maintained in a freshwater, xenic culture at 4°C. *Trypanoplasma borreli* K-100 (ATCC50432) was grown in axenic culture and was selected as a secondary outgroup.

### DNA Preparation and Sequencing

*B. saltans* genomic DNA was prepared from cell cultures after reduction of the bacterial microflora (see the Supplemental Experimental Procedures). However, the sample retained a significant bacterial component. Genomic DNA was prepared from the cell pellet using phenol-chloroform extraction and used to create 500 bp and 3 Kb genomic libraries. *T. borreli* genomic DNA was prepared directly from commercial cell culture (LGC Standards) and used to create a 500 bp genomic library. All libraries were sequenced on the Illumina HiSeq platform.

### RNA Sequencing

mRNA was purified from total RNA using an oligo-dT magnetic bead pulldown. A random-primed cDNA library was synthesized and used to create a standard Illumina library preparation with a fragment size of 400 bp. After PCR amplification, the multiplexed library was sequenced on the Illumina HiSeq 2000, resulting in 100-nt paired-end reads. Sequenced data was quality controlled and mapped to the *B. saltans* genome assembly, creating individual indexed library BAM files. Transcript abundance was estimated from BAM files using Cufflinks [42].

### Genome Assembly

To obtain the genome sequence of *B. saltans*, we applied an iterative approach. First, we corrected the reads for errors using SGA [43]. Then, 500-bp-insert sequence reads were assembled with Velvet version 1.0.18 [44], under the following parameters: kmer (41), exp_cov (auto), cov_cutoff (3), and insertsize (400). The scaffolds obtained were further joined with SSPACE [45] using first the 500-bp and then the 3-kb-insert libraries. Sequencing gaps were closed with Gapfiller [46] and Image [47]. Finally, we manually identified contigs that represented bacterial contamination and excluded reads mapping back to these. A contig was considered a contaminant if it displayed >70% similarity with the UniProt bacteria database and had no mapped RNA sequencing reads. This process was repeated three times. After the last iteration, we corrected small base errors with five iterations of ICORN [48] and split scaffolds with reads from the 3-kb-insert library and REAPR [49] under default parameters.

### Genome Annotation

The *B. saltans* genome was annotated after first screening a second time for possible bacterial contamination (see the Supplemental Experimental Procedures). Open reading frames >100 amino acids were marked up in Artemis [50]. We assumed that *B. saltans* lacks introns as all trypanosomatids genomes sequenced thus far do, and this has not subsequently been contradicted. Putative protein coding sequences were confirmed where their inferred codon usage correlated with known eukaryotic patterns, where they displayed homology with known gene products, based on BLASTp matches using BLAST2GO [51] and established protein motifs (see the Supplemental Experimental Procedures), or where their transcription was confirmed by mapping of mRNA sequencing reads.

### Proteomic Analysis

Strong anion exchange peptide fractionation and peptide analysis by online nanoflow liquid chromatography was carried out on whole-cell fractions, as well as preparations enriched for membrane proteins and glycoproteins using the nanoACQUITY-nLC system coupled to an LTQ-Orbitrap Velos mass spectrometer (see the Supplemental Experimental Procedures for full details). Tandem mass spectrometry data were searched against the predicted protein set of the *B. saltans* reference genome sequence.

### Comparative Genomics

Whole-genome alignment was carried out using the wgVISTA online tool [14]. The number of genes in the *B. saltans* genome that are co-linear with *T. brucei* was estimated, with co-linearity being defined as at least three genes with *T. brucei* orthologs arranged co-linearly with no more than two non-syntenic disruptions between each gene. Gene repertoires from the *B. saltans* and *T. borreli* draft sequences were combined with those of *T. brucei* TREU927 [8], *L. major* Friedlin [10], and the disambiguated Non-Esmeraldo gene set from *T. cruzi* CLBrenner [9]. Gene clustering was carried out using OrthoMCL 2.0 [19] with the threshold for cluster size set to maximum.

### Phylogenetics

Multiple sequence alignment was carried out using Clustalx [52] and was manually adjusted. Maximum-likelihood phylogenies were estimated using PHYML [53] with a GTR + Γ or LG + Γ model of nucleotide substitution or amino acid substitution, respectively, as appropriate. Neighbor-joining trees were estimated using MEGA [54]. Bootstrap proportions were estimated for both maximum-likelihood and neighbor-joining trees using 500 replicates. Phylogenetic reconciliation was carried out using NOTUNG [22]. Phylodiversity was estimated for 35 gene families in the conserved gene set that displayed at least three more genes in *B. saltans* than any trypanosomatid by application of the neural net and maximum-likelihood methods in Phylogenetic Diversity Analyzer [55].

### Analysis of Bodonin

Bodonin was first identified as homologous to *T. cruzi* DGF-1 protein sequences. PSI-BLAST-based comparison of these with all *B. saltans* predicted protein sequences exposed multiple gene clusters, containing both canonical and partial protein sequences. A hidden Markov model was generated from each predicted canonical bodonin sequence, and this was used to evaluate similarity with all other canonical bodonin, as well as trypanosomatid protein sets, using HMMER 3.1 [27]. Probability scores from each pairwise comparison were used to estimate a network in Cytoscape 3.2.1 [56].

## Figures and Tables

**Table 1:** Genome Size and Content Compared across Kinetoplastid Species

	*B. saltans* Konstans	*T. brucei* TREU927	*L. major* Friedlin	*T. cruzi* Non-Esmeraldo
Size (Mb)	39.9	26.1	32.8	27.8
G + C content (%)	50.9	46.4	59.7	50.7
Coding component (%)	78.9	50.5	47.9	59.8
Genes	18,943	9,068	8,272	10,834
Gene density (kb per gene)	2.1	2.9	4	2.6
Mean CDS length (bp)	1,953^a^	1,592	1,901	1,532
Median CDS length (bp)	1,467^a^	1,242	1,407	1,149
CDS G + C content (%)	53.4^a^	50.9	62.5	53.1
CEGMA score (%)^b^	79.8	78.6	78.6	68.2
Intergenic mean distance (bp)^c^	462.9	1,279	2,045	1,029
Intergenic G + C content (%)	44	41	57.3	47
Size (Mb)	39.9	26.1	32.8	27.8

CDS, coding sequence.

a Based on 10,933 complete gene models.

b See [13].

c Distance between adjacent coding sequences.

## Accession Numbers

The accession numbers for the sequence reads reported in this paper are European Nucleotide Archive: ERP000369, ERP000813, and ERP001594. The accession number for the transcriptomic data reported in this paper is ArrayExpress: E-ERAD-88. The accession number for the proteomic data reported in this paper is PRIDE: PXD002628. The accession number for the genome sequence reported in this paper is GenBank: PRJEB10421.

## Supplemental Information

Document S1. Supplemental Experimental Procedures, Figures S1–S6, and Table S1 and S2

Data S1. The Size and Identity of Kinetoplastid Gene Clusters Categorized by Phylogenetic Coverage, Related to Figure 2

Document S2. Article plus Supplemental Information
