# Supplementary material for: Kinetoplastid Phylogenomics Reveals the Evolutionary Innovations Associated with the Origins of Parasitism
Source: Curr Biol. 2016 Jan 25;26(2):161–72. doi: 10.1016/j.cub.2015.11.055 (PMC4728078; doi:10.1016/j.cub.2015.11.055)
Supplement: Document S1. Supplemental Experimental Procedures, Figures S1–S6, and Table S1 and S2 [file mmc1.pdf]

Current Biology

Supplemental Information

Kinetoplastid Phylogenomics Reveals the Evolutionary Innovations Associated with the Origins of Parasitism

Andrew P. Jackson, Thomas D. Otto, Martin Aslett, Stuart D. Armstrong, Frederic Bringaud, Alexander Schlacht, Catherine Hartley, Mandy Sanders, Jonathan M. Wastling, Joel B. Dacks, Alvaro Acosta-Serrano, Mark C. Field, Michael L. Ginger, and Matthew Berriman

Supplemental data

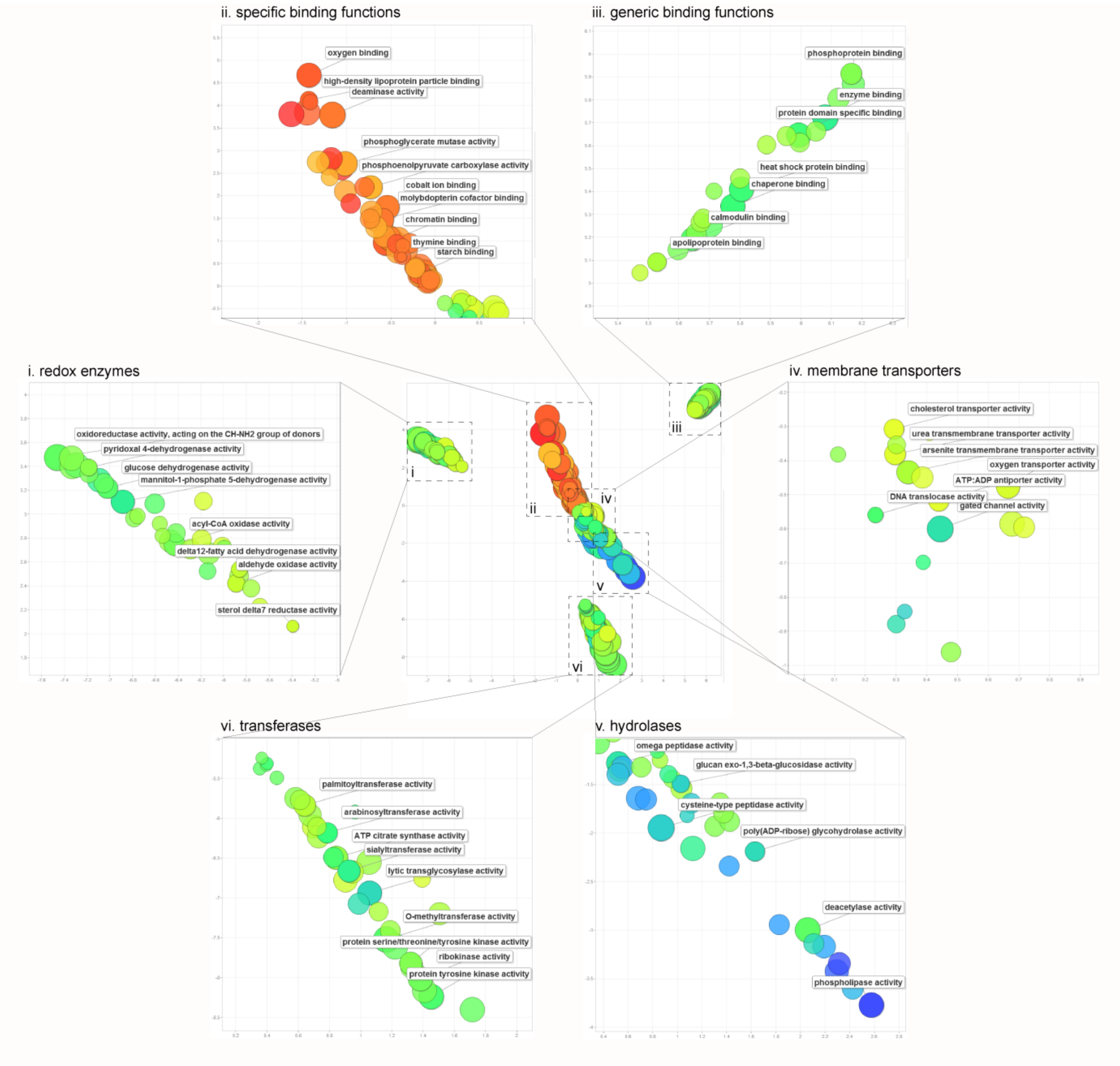

Figure S1 (related to Figure 2). Semantic clustering of Gene Ontology terms associated with the predicted proteins of 1500 genes in the ‘non-parasite’ set using REVIGO [S1]. GO terms are represented by circles. Circle size reflects the frequency of that term; circle colour reflects ‘uniqueness’, i.e. red colours denote terms with little redundancy with other terms. Semantic phrases are represented on the x and y axes. The overall clustering pattern is shown at the centre. Clusters are subdivided into six themes (i to vi) in expanded panels.

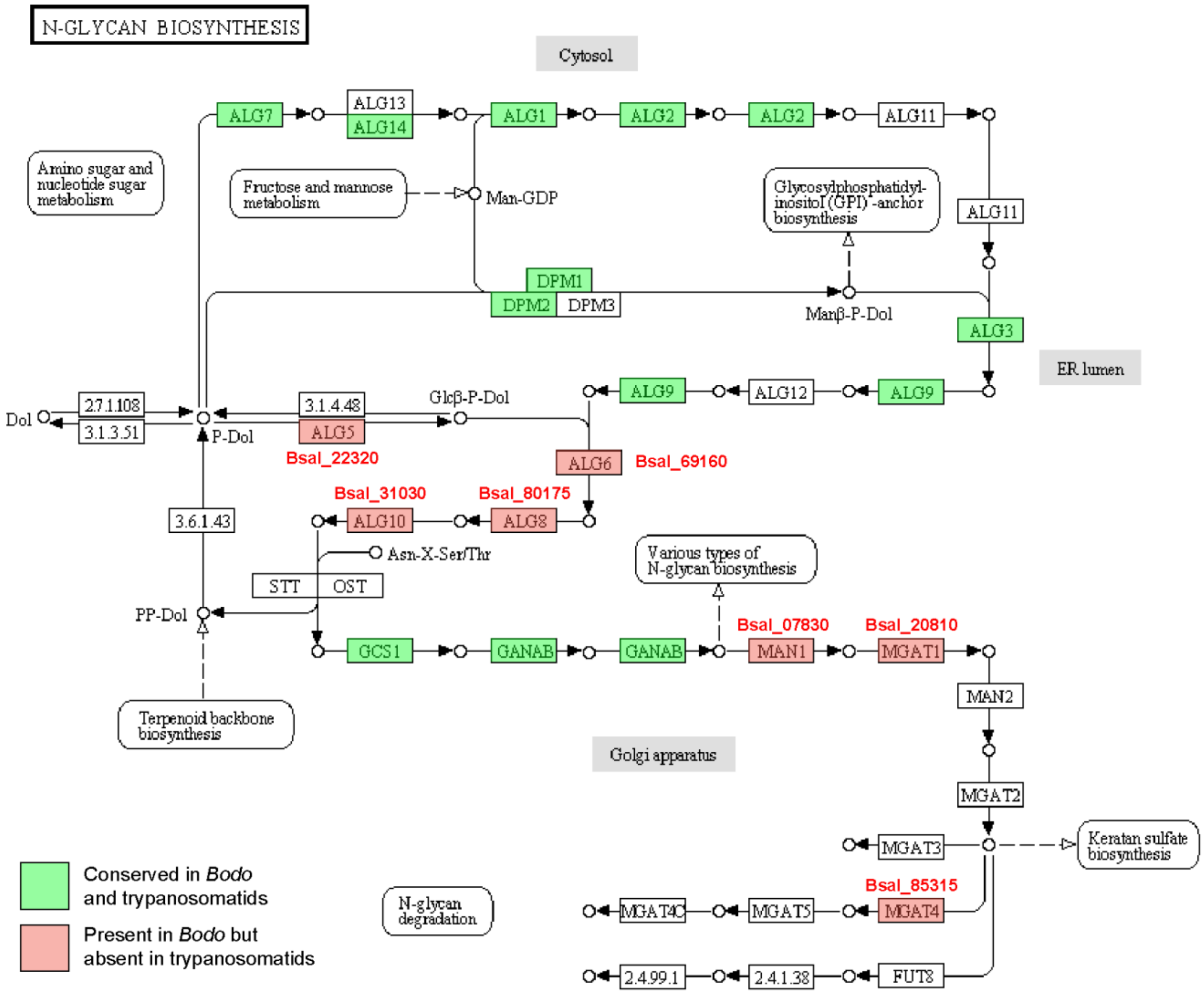

Figure S2 (related to Figure 3). Reduction of the *N*-Glycan biosynthesis pathway in trypanosomatids. The KEGG pathway for *N*-Glycan biosynthesis (K00510) is shown, with genes present in both *B. saltans* and trypanosomatids shaded green. Gene losses, i.e. components present in *B. saltans* and either *T. borreli* or another eukaryote, but absent in all trypanosomatids, are shaded red.

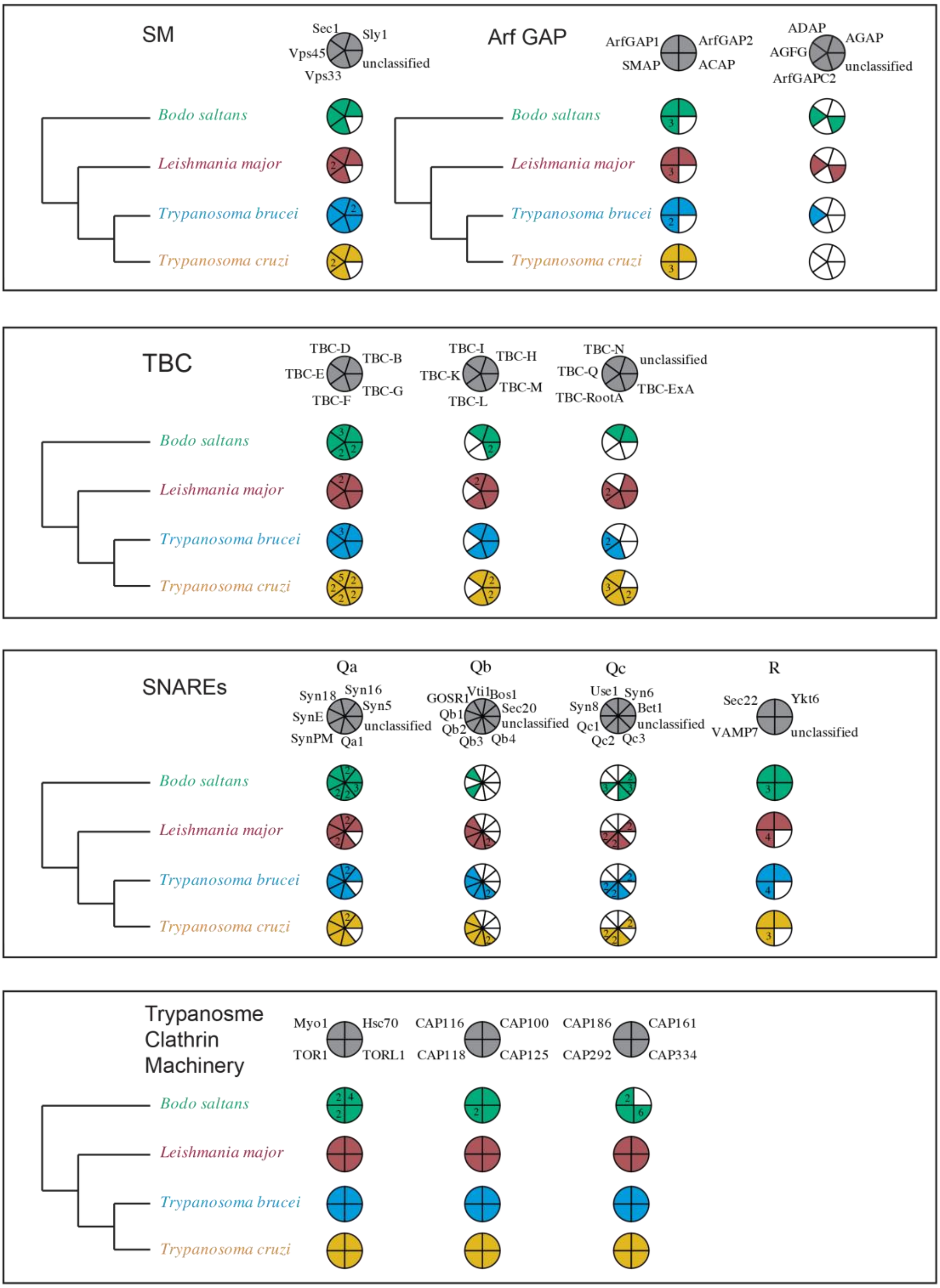

Figure S3 (related to Figure 4). Comparative genomic analysis of canonical gene families involved in membrane trafficking. Gene families are divided into family members thought to have been present in the last eukaryotic common ancestor or in excavates. BLAST and HMMER [S2] were used to identify orthologous membrane trafficking genes. Presence is denoted by coloured sectors next to the genome indicated. Empty sectors indicate that no significant orthologues were identified. If more than one paralogue was identified, the number of paralogues is indicated in the relevant sector.

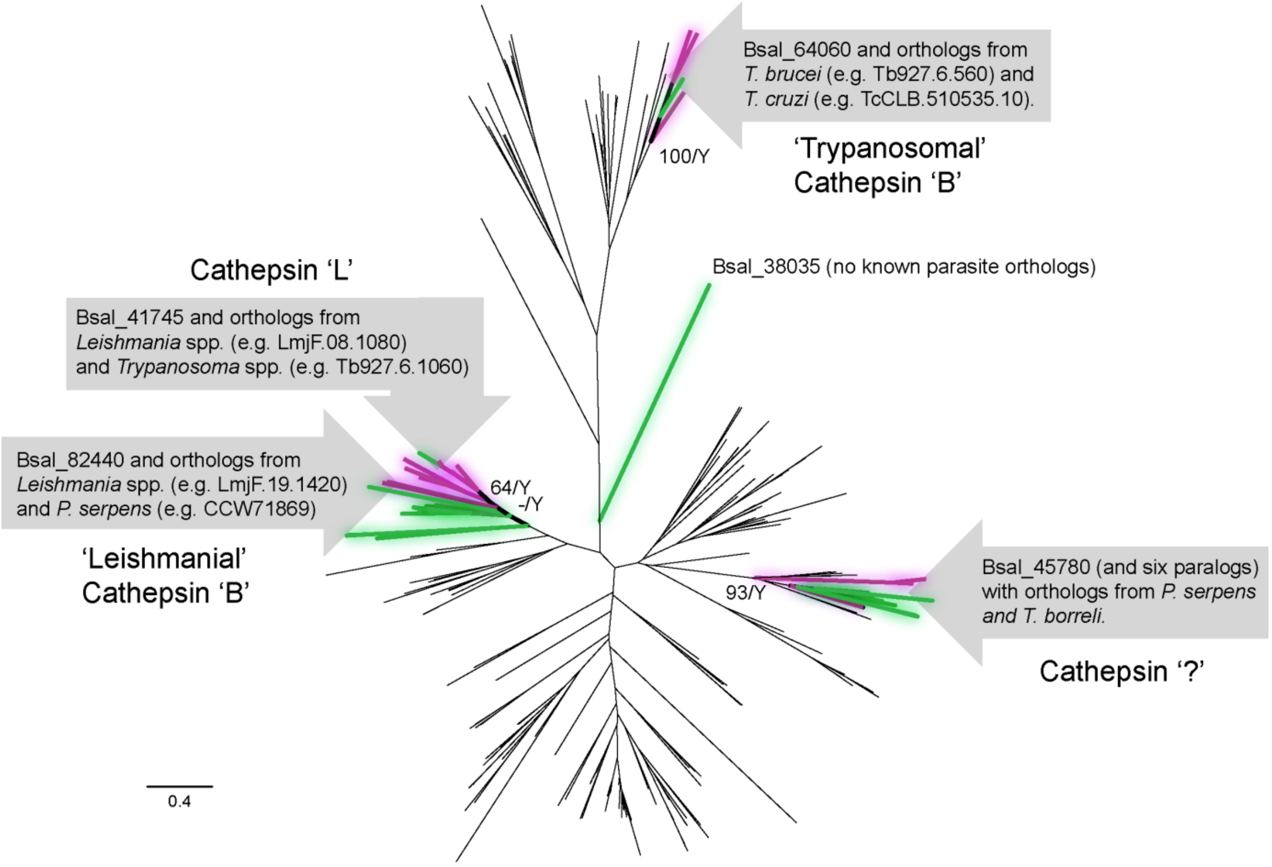

Figure S4 (related to Figure 4). Phylogenetic distribution of kinetoplastid cathepsin genes. The positions of cathepsin genes from *B. saltans* (green) and diverse trypanosomatids (*T. brucei*, *T. vivax*, *T. cruzi*, *L. major*, *L. braziliensis*, *Phytomonas serpens*, *L. pyrrhicoris* and *Crithidia deanei*; shaded pink) are shown on an unrooted maximum likelihood phylogeny. The tree includes cathepsin sequences from diverse non-kinetoplastid eukaryotes for comparison (shaded black; see methods for details). There are four distinct kinetoplastid cathepsin lineages in the tree. *B. saltans* is represented in all four clades, but no single parasite is present in all. This suggests that the ancestral cathepsin repertoire inherited by the last common ancestor of all trypanosomatids has been asymmetrically assorted among the various parasite lineages through differential gene loss.

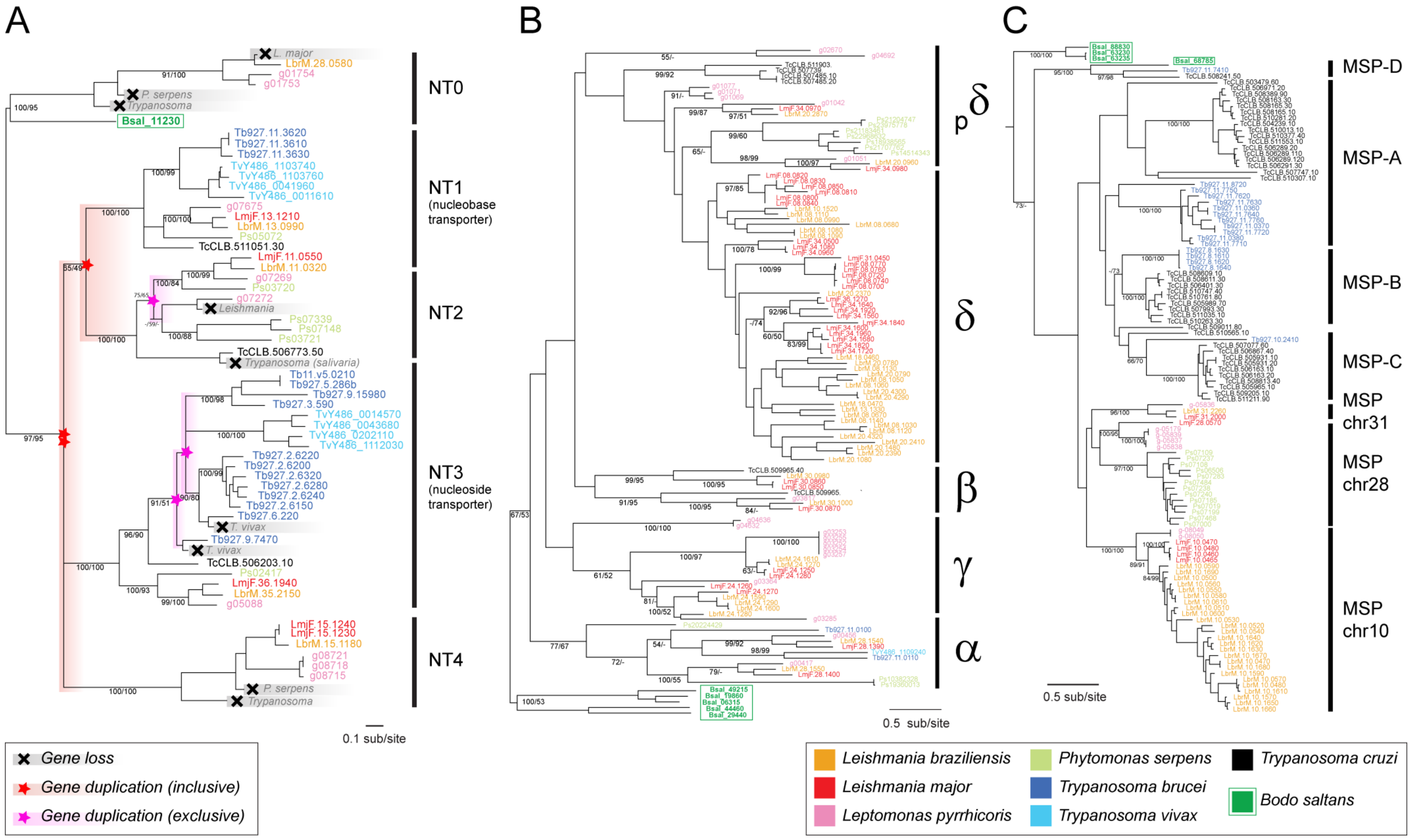
A
B
C
L. major
LbrM.28.0580
g01754
g01753
P. serpens
Trypanosoma
Bsal_11230
NT0
Tb927.11.3620
Tb927.11.3610
Tb927.11.3630
TvY486_1103740
TvY486_1103760
TvY486_0041960
TvY486_0011610
g07675
LmjF.13.1210
LbrM.13.0990
Ps05072
TcCLB.511051.30
NT1
(nucleobase
transporter)
LmjF.11.0550
LbrM.11.0320
g07269
Ps03720
g07272
Leishmania
Ps07339
Ps07148
Ps03721
TcCLB.506773.50
Trypanosoma (salivaria)
NT2
Tb11.v5.0210
Tb927.5.286b
Tb927.9.15980
Tb927.3.590
TvY486_0014570
TvY486_0043680
TvY486_0202110
TvY486_1112030
Tb927.2.6220
Tb927.2.6200
Tb927.2.6320
Tb927.2.6280
Tb927.2.6240
Tb927.2.6150
Tb927.6.220
T. vivax
Tb927.9.7470
T. vivax
TcCLB.506203.10
Ps02417
LmjF.36.1940
LbrM.35.2150
g05088
NT3
(nucleoside
transporter)
LmjF.15.1240
LmjF.15.1230
LbrM.15.1180
g08721
g08718
g08715
P. serpens
Trypanosoma
NT4
0.1 sub/site
pδ
δ
β
γ
α
0.5 sub/site
MSP-D
MSP-A
MSP-B
MSP-C
MSP
chr31
MSP
chr28
MSP
chr10
0.5 sub/site
Gene loss
Gene duplication (inclusive)
Gene duplication (exclusive)
Leishmania braziliensis
Leishmania major
Leptomonas pyrrhicoris
Phytomonas serpens
Trypanosoma brucei
Trypanosoma vivax
Trypanosoma cruzi
Bodo saltans

Figure S5 (related to Figure 5). Principal parasite-specific gene family innovations. (A) Maximum likelihood phylogeny of nucleoside transporter genes estimated from amino acid sequences using a LG+Γ model. Terminal nodes are labelled with gene identifiers and shaded according to species. Clades are labelled at right according to conserved function or genomic position. Gene duplications and losses are inferred following reconciliation with a species phylogeny. Bootstrap values for maximum likelihood (left) and neighbour-joining (right) analyses are shown below subtending branches. The tree is rooted using the clade containing the single *B. saltans* homolog. **(B)** Maximum likelihood phylogeny of amastin glycoprotein genes estimated from amino acid sequences using a LG+Γ model. Terminal nodes are labelled with gene identifiers and shaded according to species. Clades are labelled at right according to a previously published classification [S3]. The tree is rooted using a clade containing *B. saltans* homologs. **(C)** Maximum likelihood phylogeny of Major Surface Protease (MSP) genes estimated from amino acid sequences using a LG+Γ model. Terminal nodes are labelled with gene identifiers and shaded according to species. Clades are labelled at right according to a previously published classification [S4]. The tree is rooted using the clade containing *B. saltans* homologs. The progenitor of parasite-specific derivations can be identified as the single-copy MSP-D locus, which is orthologous with Bsal_68785.

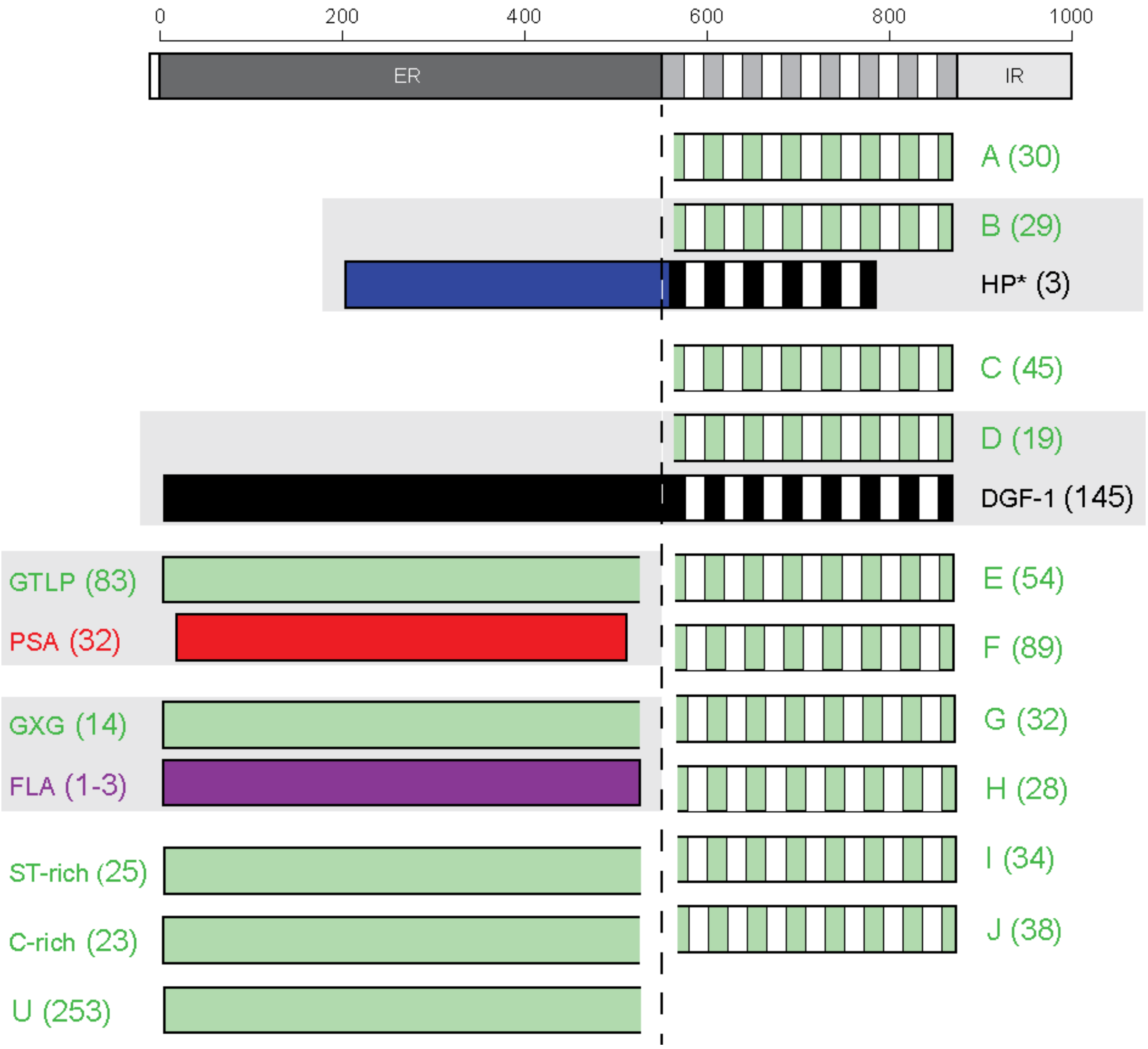

Figure S6 (related to Figure 6). Genomic repertoire of bodonin predicted proteins. In this cartoon, shaded rectangles represent protein sequences. The canonical structure of bodonin is shown at the top in grey, with a scale bar measured in amino acid residues. The structure consists of a membrane-spanning region, including seven transmembrane domains (in white), preceded by an extracellular region (ER; dark grey) and followed by a shorter intracellular region (IR; light grey). Beneath the canonical structure are shown several sub-groups of homologous proteins. Those shaded green are predicted proteins of *B. saltans* genes; these can be sorted into 10 transmembrane region types (A-J) based on phylogenetic relationships (data not shown). The number of each in the *B. saltans* genome is given in parentheses. Alternatively, *B. saltans* homologs can be classified according to the composition of the ER. The two classifications do not necessarily agree, i.e. ER and transmembrane region identities are decoupled. Additionally, bodonin-like proteins from trypanosomatids are represented. A hypothetical protein from *T. cruzi*, marked with an asterisk, is shown in blue; this is most closely related to bodonin clade B. DGF-1 proteins from *T. cruzi* are most closely related to bodonin clade D. PSA proteins from *Leishmania* spp. lack a transmembrane region but possess an amino acid repeat sequences almost identical to 'GTLP'-type bodonin. Finally, the flagellar attachment zone protein FLA1 in *T. brucei* (and its orthologs in other species) is shaded purple. FLA1 also lacks a transmembrane region but shares the same repeat structure as 'GXG'-type bodonin.

Table S1 (related to Figure 3). Results of mapping the 'non-parasite' gene set against metabolic pathways using the KEGG Ortholog (KO) database. The pathways shown display an excess of genes in *B. saltans*, relative to trypanosomatids collectively, which is expressed as a percentage.

**KEGG pathway**	**Number of genes in:**		**% increase in *B. saltans***
	non-parasite gene set *	conserved gene set**	
00010 Glycolysis / Gluconeogenesis	3	20	15
00020 Citrate cycle	2	13	15
00030 Pentose phosphate pathway	1	13	8
00040 Pentose and glucuronate interconversions	6	2	300
00051 Fructose and mannose metabolism	2	10	20
00052 Galactose metabolism	2	5	40
00053 Ascorbate and aldarate metabolism	4	2	200
00061 Fatty acid biosynthesis	1	2	50
00062 Fatty acid elongation	4	3	133
00071 Fatty acid metabolism	8	6	133
00100 Steroid biosynthesis	2	5	40
00130 Ubiquinone and other terpenoid-quinone biosynthesis	2	2	100
00140 Steroid hormone biosynthesis	2	1	200
00230 Purine metabolism	16	38	42
00240 Pyrimidine metabolism	8	35	23
00250 Alanine, aspartate and glutamate metabolism	2	13	15
00260 Glycine, serine and threonine metabolism	6	9	67
00270 Cysteine and methionine metabolism	2	16	13
00280 Valine, leucine and isoleucine degradation	6	11	55
00281 Geraniol degradation	1	3	33
00300 Lysine biosynthesis	3	1	300
00310 Lysine degradation	6	3	200
00330 Arginine and proline metabolism	8	10	80
00340 Histidine metabolism	3	1	300
00350 Tyrosine metabolism	7	6	117
00360 Phenylalanine metabolism	3	3	100
00380 Tryptophan metabolism	8	4	200
00400 Phenylalanine, tyrosine and tryptophan biosynthesis	1	3	33
00401 Novobiocin biosynthesis	1	2	50
00410 beta-Alanine metabolism	5	5	100
00430 Taurine and hypotaurine metabolism	1	1	100
00450 Selenocompound metabolism	1	5	20
00460 Cyanoamino acid metabolism	1	4	25
00480 Glutathione metabolism	1	16	6
00500 Starch and sucrose metabolism	5	6	83
00510 N-Glycan biosynthesis	9	11	82
00511 Other glycan degradation	3	1	300
00513 Various types of N-glycan biosynthesis	7	5	140
00520 Amino sugar and nucleotide sugar metabolism	4	17	24
00524 Butirosin and neomycin biosynthesis	2	2	100
00531 Glycosaminoglycan degradation	1	0	100
00561 Glycerolipid metabolism	6	7	86
00562 Inositol phosphate metabolism	7	10	70
00563 Glycosylphosphatidylinositol	2	7	29
00564 Glycerophospholipid metabolism	7	11	64
00565 Ether lipid metabolism	4	4	100
00590 Arachidonic acid metabolism	3	2	150
00592 alpha-Linolenic acid metabolism	2	1	200
00600 Sphingolipid metabolism	1	0	100
00600 Sphingolipid metabolism	3	4	75
00601 Glycosphingolipid biosynthesis - lacto and neolacto	2	0	200
00603 Glycosphingolipid biosynthesis - globo series	1	0	100
00620 Pyruvate metabolism	4	14	29
00624 Polycyclic aromatic hydrocarbon degradation	1	2	50
00630 Glyoxylate and dicarboxylate metabolism	1	8	13
00640 Propanoate metabolism	4	8	50
00643 Styrene degradation	3	1	300
00650 Butanoate metabolism	2	6	33
00670 One carbon pool by folate	1	5	20
00720 Carbon fixation pathways in prokaryotes	2	3	67
00730 Thiamine metabolism	1	1	100

00750 Vitamin B6 metabolism	2	2	100
00760 Nicotinate and nicotinamide metabolism	3	5	60
00770 Pantothenate and CoA biosynthesis	3	3	100
00780 Biotin metabolism	1	2	50
00790 Folate biosynthesis	2	4	50
00791 Atrazine degradation	1	1	100
00900 Terpenoid backbone biosynthesis	1	0	100
00900 Terpenoid backbone biosynthesis	1	7	14
00910 Nitrogen metabolism	1	10	10
00920 Sulfur metabolism	1	3	33
00930 Caprolactam degradation	1	3	33
00950 Isoquinoline alkaloid biosynthesis	2	3	67
00960 Tropane, piperidine and pyridine alkaloid biosynthesis	1	3	33
00970 Aminoacyl-tRNA biosynthesis	2	21	10
00980 Metabolism of xenobiotics by cytochrome P450	1	0	100
00981 Insect hormone biosynthesis	3	0	300
00983 Drug metabolism - other enzymes	4	6	67
01040 Biosynthesis of unsaturated fatty acids	3	2	150
01051 Biosynthesis of ansamycins	1	1	100
01053 Biosynthesis of siderophore group nonribosomal peptides	3	0	300
02010 ABC transporters	14	2	700
02020 Two-component system	1	6	17
03008 Ribosome biogenesis in eukaryotes	3	40	8
03013 RNA transport	4	30	13
03015 mRNA surveillance pathway	2	22	9
03018 RNA degradation	4	21	19
03022 Basal transcription factors	1	6	17
03030 DNA replication	1	23	4
03040 Spliceosome	4	32	13
03050 Proteasome	1	29	3
03060 Protein export	1	9	11
03320 PPAR signaling pathway	6	5	120
03410 Base excision repair	5	13	38
03420 Nucleotide excision repair	3	19	16
03430 Mismatch repair	1	13	8
03440 Homologous recombination	3	10	30
03450 Non-homologous end-joining	2	4	50
03460 Fanconi anemia pathway	1	10	10
04010 MAPK signaling pathway	19	6	317
04011 MAPK signaling pathway - yeast	2	1	200
04012 ErbB signaling pathway	6	3	200
04013 MAPK signaling pathway - fly	2	1	200
04020 Calcium signaling pathway	10	8	125
04062 Chemokine signaling pathway	6	3	200
04064 NF-kappa B signaling pathway	2	2	100
04066 HIF-1 signaling pathway	8	11	73
04070 Phosphatidylinositol signaling system	10	10	100
04080 Neuroactive ligand-receptor interaction	1	1	100
04110 Cell cycle	4	25	16
04111 Cell cycle - yeast	5	30	17
04113 Meiosis - yeast	4	24	17
04114 Oocyte meiosis	8	20	40
04115 p53 signaling pathway	2	2	100
04120 Ubiquitin mediated proteolysis	14	21	67
04122 Sulfur relay system	1	5	20
04130 SNARE interactions in vesicular transport	1	13	8
04141 Protein processing in endoplasmic reticulum	18	30	60
04142 Lysosome	12	15	80
04144 Endocytosis	9	23	39
04145 Phagosome	9	21	43
04146 Peroxisome	7	12	58
04150 mTOR signaling pathway	5	7	71
04151 PI3K-Akt signaling pathway	10	11	91
04210 Apoptosis	3	6	50
04260 Cardiac muscle contraction	8	3	267
04270 Vascular smooth muscle contraction	11	4	275
04310 Wnt signaling pathway	4	8	50
04320 Dorso-ventral axis formation	3	1	300
04330 Notch signaling pathway	2	2	100
04350 TGF-beta signaling pathway	4	5	80

04360 Axon guidance	6	3	200
04370 VEGF signaling pathway	5	4	125
04380 Osteoclast differentiation	6	4	150
04390 Hippo signaling pathway	1	6	17
04391 Hippo signaling pathway -fly	2	5	40
04510 Focal adhesion	6	3	200
04520 Adherens junction	3	2	150
04530 Tight junction	4	4	100
04540 Gap junction	6	4	150
04612 Antigen processing and presentation	3	3	100
04614 Renin-angiotensin system	1	2	50
04620 Toll-like receptor signaling pathway	2	2	100
04621 NOD-like receptor signaling pathway	3	3	100
04622 RIG-I-like receptor signaling pathway	2	2	100
04650 Natural killer cell mediated cytotoxicity	4	4	100
04660 T cell receptor signaling pathway	5	4	125
04662 B cell receptor signaling pathway	5	4	125
04664 Fc epsilon RI signaling pathway	3	2	150
04666 Fc gamma R-mediated phagocytosis	6	5	120
04668 TNF signaling pathway	3	4	75
04670 Leukocyte transendothelial migration	3	1	300
04710 Circadian rhythm	2	3	67
04713 Circadian entrainment	8	3	267
04720 Long-term potentiation	6	6	100
04722 Neurotrophin signaling pathway	6	5	120
04723 Retrograde endocannabinoid signaling	7	3	233
04724 Glutamatergic synapse	6	5	120
04725 Cholinergic synapse	7	3	233
04726 Serotonergic synapse	9	2	450
04727 GABAergic synapse	7	4	175
04742 Taste transduction	1	1	100
04744 Phototransduction	3	1	300
04745 Phototransduction - fly	2	1	200
04810 Regulation of actin cytoskeleton	7	6	117
04910 Insulin signaling pathway	9	11	82
04911 Insulin secretion	9	1	900
04912 GnRH signaling pathway	13	3	433
04915 Estrogen signaling pathway	6	7	86
04916 Melanogenesis	5	3	167
04920 Adipocytokine signaling pathway	5	4	125
04960 Aldosterone-regulated sodium reaBsal_orption	3	2	150
04961 Endocrine and other factor-regulated calcium	2	5	40
04962 Vasopressin-regulated water reaBsal_orption	4	6	67
04964 Proximal tubule bicarbonate reclamation	1	1	100
04970 Salivary secretion	6	3	200
04971 Gastric acid secretion	3	2	150
04972 Pancreatic secretion	2	3	67
04973 Carbohydrate digestion and aBsal_orption	7	2	350
04975 Fat digestion and aBsal_orption	3	2	150
04976 Bile secretion	11	2	550
05100 Bacterial invasion of epithelial cells	2	3	67
05110 Vibrio cholerae infection	4	15	27
05130 Pathogenic Escherichia coli infection	1	3	33
05131 Shigellosis	2	4	50
05132 Salmonella infection	5	5	100
05133 Pertussis	3	2	150
05134 Legionellosis	4	11	36
05140 Leishmaniasis	2	3	67
05142 Chagas disease	1	8	13
05143 African trypanosomiasis	1	0	100
05145 Toxoplasmosis	4	4	100
05146 Amoebiasis	3	3	100

* Present in *B.* saltans and T. *borreli*, but not trypanosomatids.
****** Present in *B. saltans* and one or more trypanosomatid genome.

Table S2 (related to Figure 4). Phylogenetic diversity (PD) of conserved gene families across four species. The number of individual genes and distinct phylogenetic lineages is noted. PD is calculated using nearest-neighbor (NN) and maximum likelihood (ML) methods. The percentage reduction in trypanosomatids collectively relative to *B. saltans* is calculated for both lineages and PD.

Gene family	Gene number:					Lineage number:				Phylodiversity (NN)				Phylodiversity (ML)				% reduction	
	Bs	*Tb*	*Lm*	*Tc*	mean	*Bs*	*Tb*	*Lm*	*Tc*	*Bs*	*Tb*	*Lm*	*Tc*	*Bs*	*Tb*	*Lm*	*Tc*	Lineages	PD
lysosomal membrane protein p67	12	2	0	0	0.7	5	1	0	0	3.52	0.01	-	-	10.82	2.76	-	-	93.33	99.81
acid phosphatase	7	0	0	1	0.3	4	0	0	1	2.07	-	-	0.00	10.18	-	-	3.12	91.67	99.78
WD40 repeat-containing protein	9	1	2	1	1.3	6	1	1	1	2.97	-	0.14	-	9.82	-	1.96	-	83.33	95.12
ferric reductase	7	1	1	1	1.0	4	1	1	1	2.21	-	0.17	-	17.21	-	3.65	-	75.00	92.49
endomembrane protein 70	12	0	2	2	1.3	7	0	2	1	3.76	-	0.79	0.03	12.57	-	4.87	0.04	85.71	89.04
hypothetical protein	15	3	2	1	2.0	8	2	2	1	5.64	0.68	0.61	-	24.18	3.47	3.43	-	79.17	88.60
ammonium transporter	6	0	0	1	0.3	3	0	0	1	1.40	-	-	0.24	7.67	-	-	0.36	88.89	83.06
PLAC8 domain-containing protein	36	0	2	0	0.7	7	0	2	0	9.62	-	1.68	-	32.28	-	10.43	-	90.48	82.57
ion channel protein-like	14	1	2	0	1.0	7	1	2	0	4.34	-	0.81	-	17.37	-	4.60	-	85.71	81.28
voltage-gated calcium ion channel	16	1	1	1	1.0	10	1	2	1	4.81	-	1.03	-	23.12	-	8.88	-	86.67	78.56
lysophospholipase	4	1	1	1	1.0	3	1	1	1	1.29	0.36	-	-	421.35	240.66	-	-	66.67	71.84
NADP-dependent alcohol dehydrogenase	4	0	1	1	0.7	2	0	1	1	0.49	-	0.16	-	0.92	-	0.20	-	66.67	67.21
cathepsin cysteine protease	22	12	8	9	9.7	7	2	3	2	5.53	2.72	1.09	1.76	17.21	6.15	4.32	6.42	66.67	66.44
lipase	15	4	5	7	5.3	5	3	3	3	4.48	1.23	1.52	1.87	23.10	11.75	8.56	12.45	40.00	65.68
beta-lactamase related protein	4	0	1	1	0.7	3	0	1	1	1.15	-	0.40	-	3.81	-	3.01	-	77.78	65.40
ABC transporter	66	16	32	17	21.7	36	22	22	22	16.27	5.49	8.89	5.18	52.44	25.36	31.28	24.70	38.89	59.92
solute carrier protein	5	1	2	1	1.3	5	1	2	1	1.98	-	0.79	-	7.15	-	3.89	-	73.33	59.80
mannosyltransferase-like protein	7	0	2	1	1.0	4	0	1	1	1.89	-	0.86	-	8.83	-	4.06	-	83.33	54.18
phosphodiesterase	9	5	4	5	4.7	7	3	5	4	2.73	1.02	1.76	1.45	13.00	8.82	13.70	11.77	42.86	48.42
dual-specificity protein phosphatase	15	5	10	14	9.7	12	9	6	9	5.25	3.87	1.79	2.60	30.20	23.08	8.32	10.50	33.33	47.61
hypothetical protein	6	0	2	4	2.0	6	0	2	4	2.25	-	0.79	1.58	9.03	-	5.49	8.98	66.67	47.30
short chain dehydrogenase	27	11	22	12	15.0	18	13	15	13	8.92	4.09	6.04	4.02	30.55	18.56	25.42	17.66	24.07	47.14
actin-like proteins	14	8	6	7	7.0	13	7	6	7	4.70	3.23	2.24	2.44	20.75	11.50	11.15	11.16	48.72	43.88
dynein heavy chain, cytosolic	20	14	14	7	11.7	16	14	14	14	6.71	4.61	4.90	2.26	43.75	30.02	41.19	20.60	12.50	41.57
endosomal integral membrane protein	12	4	4	3	3.7	8	5	5	5	1291.09	779.25	901.02	630.45	1291.09	950.48	1072.24	801.68	37.50	40.34
kinesin K39-like	13	5	11	6	7.3	13	5	7	5	3.13	1.35	2.73	1.62	11.99	4.65	8.43	5.38	56.41	39.23
phospholipase c-like protein	5	1	3	1	1.7	5	1	3	1	1.48	-	0.90	-	5.86	-	3.99	-	66.67	39.15
EF hand domain-containing protein	8	5	4	5	4.7	8	5	5	5	2.85	1.88	1.52	1.88	18.99	14.09	9.75	13.21	37.50	38.16
P-type ATPase	24	14	16	8	12.7	16	11	13	11	3.12	2.52	2.56	1.14	16.32	14.41	16.77	11.88	27.08	33.61
GTP-binding protein	54	38	37	38	37.7	16	15	13	13	61.69	51.24	49.68	44.29	63.70	41.74	51.76	46.37	14.58	21.54
RNA-binding protein	79	59	67	61	62.3	66	53	54	53	ND	ND	ND	ND	87.50	65.50	77.14	65.01	19.19	20.90

Supplemental Experimental Procedures

Bacterial reduction during DNA preparation. Broad spectrum antibiotics were applied twelve hours prior to extraction. Differential centrifugation was used to promote separation of *B. saltans* from the bacterial microflora. Centrifugation of the culture at 3,000rpm for 25 minutes succeeded in pelleting all protists, leaving many bacterial cells in the supernatant. Finally, passing the remaining pellet through a Percoll gradient further reduced the bacterial component. Despite these measures, the cell pellet retained significant bacterial contamination, not least from within the living bodonids.

***Trypanoplasma* genome assembly**. The *T. borreli* genome was assembled using Velvet as described in the main text. Initial attempts at assembly were unsuccessful and demonstrated that a complex repeat within the genome was inhibiting good results. The final assembly excluded reads that mapped to this complex repeat.

RNA sequencing. Total RNA was isolated using an RNeasy Mini Kit (Qiagen). RNA quality and the relative contributions of total and small RNA were assessed by the Agilent 2100 Bioanalyzer microfluidics-based platform (Agilent Technologies, Santa Clara, USA). Poly-adenylated RNA (mRNA) was purified from total RNA using an oligo-dT magnetic bead pull-down, using TruSeq RNA Sample Prep v2 kits (Illumina). The mRNA was then fragmented using metal ion-catalyzed hydrolysis. A random-primed cDNA library was synthesized and double-strand cDNA was used as the input to a standard Illumina library preparation, with 400bp fragment size. The libraries were amplified with 10 cycles of PCR using KAPA Hifi Polymerase. Samples were quantified and pooled based on a post-PCR Agilent Bioanalyzer, followed by size-selection using the LabChip XT Caliper. The multiplexed library was sequenced on the Illumina HiSeq 2000 with forward and reverse primers, according to the manufacturers standard protocol, resulting in 100-nucleotide paired-end reads.

Sample preparation for proteomics. To solubilise protein, *B. saltans* cell pellets were sonicated in 50 mM ammonium bicarbonate (Sigma-Aldrich) and 0.1% (w/v) Rapigest (Waters, Elstree, UK) on ice. Samples were then incubated at 80 °C for 10 min and reduced with 3 mM dithiothreitol (DTT) at 60°C for 10 minutes then alkylated with 9 mM iodoacetimde at room temperature for 30 minutes in the dark. Proteomic-grade trypsin (Sigma-Aldrich) was added at a protein:trypsin ratio of 50:1, and samples were incubated at 37 °C overnight before removal of Rapigest by trifluoroacetic acid precipitation.

Strong anion exchange peptide fractionation and peptide analysis by online nanoflow liquid chromatography using the nanoACQUITY-nLC system (Waters) coupled to an LTQ-Orbitrap Velos (ThermoFisher Scientific, Bremen, Germany) mass spectrometer was performed as previously described [S5]. For samples run on the Q-Exactive platform (Thermo Fisher Scientific), peptides were loaded on a 50cm Easy-Spray column with an internal diameter of 75µm, packed with 2µm C18 particles, fused to a silica nano-electrospray emitter (Thermo Fisher Scientific). Reversed phase liquid chromatography was performed using the Thermo EASY-nLC 1000 with a binary buffer system consisting of 0.1% formic acid (buffer A) and 80% acetonitrile in 0.1% formic acid (buffer B). The peptides were separated by a linear gradient of 3.8 – 50% buffer B over 247 min at a flow rate of 300nl/min. The column was operated at a constant temperature of 35°C.The Q-Exactive was operated in data-dependent mode with survey scans acquired at a resolution of 70,000 at m/z 200. Up to the top 10 most abundant isotope patterns with charge states +2, +3 and/or +4 from the survey scan were selected with an isolation window of 2.0Th and fragmented by higher energy collisional dissociation with normalized collision energies of 30. The maximum ion injection times for the survey scan and the MS/MS scans were 250 and 100ms, respectively, and the ion target value was set to $1.0e^6$ for survey scans and $1.0e^4$ for the MS/MS scans.

Proteomic analysis. Spectral data were analysed using the PEAKS studio 7 software (Bioinformatics Solutions Inc., Waterloo, ON, Canada). Tandem MS data were searched against the predicted protein set of the *B. saltans* reference genome sequence. Search parameters were as follows; precursor mass tolerance set to 10ppm and fragment mass tolerance set to 0.8 Da or 0.01 Da. One missed tryptic cleavage was permitted. Carbamidomethylation (cysteine) was set as a fixed modification and oxidation (methionine) set as a variable modification. The false discovery rate was set at 1%.

Membrane protein enrichment. Protein extraction and Triton X-114 partitioning was performed as described [S6]. Acetone precipitated proteins were resuspended in SDS-PAGE reducing sample buffer and were run approximately 1 cm into a NuPAGE® (Life Technologies) precast 4-12% gel Tris-Bis gradient gel and the proteins were stained with colloidal Coomassie Blue G-250 (Simply BlueTM, Life Technologies) according to the manufacturer's instructions. The entire gel lane was excised and cut into smaller pieces (approx. 1mm3) and in-gel tryptic digestion performed as described elsewhere [S7].

Glycoprotein enrichment. *B. saltans* cell pellets were solubilised in 8M urea, 4.5 M thiourea, 2.5 % (w/v) CHAPS, 50mM Tris-HCL pH 7.4, 66mM DTT. N-linked glycan enrichment was performed using Concanavilin A spin columns (Thermo Scientific Pierce Glycoprotein Isolation Kit) according to the manufacturer's instructions. Eluate containing glycans was precipitated by incubation with 30% Trichloroacetic acid (w/v) in acetone for 2 hours on ice and the pellet was resuspended in SDS-PAGE sample buffer. SDS-PAGE and in-gel tryptic digestion was performed as above.

Genome annotation. All assembled contigs were analyzed with BLASTx; to be included in the assembly, a contig had to include an ORF >100 amino acids in length with a best match to a known trypanosomatid protein, or otherwise be homologous to ORFs physically linked to known trypanosomatids proteins in other contigs. In practice, there were many contigs with no good matches to existing databases, which were likely to be *Bodo*-specific; many of these were accepted because they included multi-copy genes found adjacent to known trypanosomatid genes elsewhere. In addition, we required all contigs to be represented in transcriptomic data; six contigs that lacked BLAST matches were removed because they also lacked any RNA-seq coverage. PfamScan and InterproScan 5.0 [S8]. SignalP 4.1 [S9], TMHMM 2.0 [S10] and Fraganchor [S11] were used to predict signal peptides, transmembrane helices and GPI-anchors across the genome. Non-coding RNA genes were identified using Rfam [S12]. Sequence repeats were annotated using custom perl scripts.

Whole genome alignment. *B. saltans* contigs were first sorted into bins based on *T. brucei* chromosome. BLASTx was used to determine which *T. brucei* chromosome each *B. saltans* contig had most affinity with. For large contigs with multiple regions of distinct affinities, the *B. saltans* sequence was split. *B. saltans* contigs in each bin were then aligned with the appropriate *T. brucei* chromosome using wgVISTA [S13], with the option for translated anchoring applied.

Gene clustering. The *T. borreli* sequence is included to allow us to distinguish between genes that are present in *B. saltans* but absent from all trypanosomatids because they have been lost in the parasites from those that have evolved in *B. saltans* uniquely. *Trypanoplasma* (*Cryptobia*) *borreli* is a parabodonid parasite of fish. As *B. saltans* is more closely related to trypanosomatids than *T. borreli*, any genes shared by both *B. saltans* and *T. borreli* but absent from trypanosomatids will most likely represent a gene loss in the latter, rather than a *Bodo*-specific gene gain.

The resultant clusters were sorted according to their taxonomic distribution into conserved and taxon-specific cohorts. Extensive manual curation was applied using BLASTp to check all putative species-specific clusters to ensure that no distant homologs existed in other genomes, and to confirm putative gene losses from conserved gene clusters. Single-copy genes not included in any clusters were extracted and compared using reciprocal BLASTp against all other protein sets to confirm that they were species-specific.

6444 *B. saltans* genes, or 34.0%, were species-specific with no detectable homology with any other sequence in any database. Although these may also represent losses in trypanosomatids, this cannot currently be confirmed due to lack of additional genome data and we assume that these genes evolved uniquely in eubodonids.

Phylogenetic analysis. Reconciliation analysis was carried out using NOTUNG, with the species tree defined as and the species tree [[[[*T. brucei, T. vivax*], *T. cruzi*], [[[*L. major, L. braziliensis*], *Leptomonas pyrrhocoris*], *Phytomonas serpens*]], *B. saltans*]. Phylodiversity was estimated for 35 gene families in the conserved gene set that displayed at least three more genes in *B. saltans* than any trypanosomatid. Homologs for each gene family from *B. saltans*, *T. brucei*, *T. cruzi* and *L. major* were combined with homologs from a selection of non-kinetoplastids that are representative of eukaryotic diversity. Non-kinetoplastid homologs were mined from the respective genome databases of *Acanthamoeba castellani, Arabidopsis thaliana, Tetrahymena thermophila, Paramecium caudatum, Aspergillus fumigatus* and *Naegleria fowleri* using BLASTp. Neighbour-joining trees were estimated from multiple sequence alignments of each gene family using MEGA [S14]. The phylodiversity (PD [S15]) of each gene family in *B. saltans* was calculated by applying the NN and ML methods in Phylogenetic Diversity Analyzer (PDA [S16]) to the tree with the trypanosomatid sequences masked. This was repeated for *T. brucei*, *T. cruzi* and *L. major* in turn, in each case calculating PD from a tree containing the trypanosomatid concerned and non-kinetoplastid outgroups, but with other kinetoplastids masked. This process produced NN and ML estimates of PD for each Kinetoplastid, allowing a percentage reduction in each parasite relative to *B. saltans* to be calculated.

Analysis of bodonin. Alignment of canonical bodonin sequences, as defined in Figure S6, relied on the membrane-spanning region only, since this region alone was homologous across all copies. Based on the

resultant neighbor-joining phylogeny, the canonical repertoire was segregated into clades A-J. Predictions of glycosylation sites on canonical bodonin protein sequences were made using GPP [S17] and NetNGlyc 1.0/NetOGlyc 4.0 [S18]. Comparison of bodonin extracellular regions to protein tertiary structure databases was carried out with pGenThreader [S19], within the PsiPred platform [S20].

Supplemental References

S1. Supek, F., Bošnjak, M., Škunca, N. and Šmuc, T. (2011). REVIGO summarizes and visualizes long lists of gene ontology terms. PLoS One *6*, e21800.

S2. Zhang, Z. and Wood, W.I. (2003). A profile hidden Markov model for signal peptides generated by HMMER. Bioinformatics *19*, 307-308.

S3. Jackson, A.P. (2010). The evolution of amastin surface glycoproteins in trypanosomatid parasites. Mol Biol Evol *27*, 33-45.

S4. Yao, C. (2010). Major surface protease of trypanosomatids: one size fits all? Infect Immun *78*, 22-31.

S5. Darby, A.C., Gill, A.C., Armstrong, S.D., Hartley, C.S., Xia, D., Wastling, J.M. and Makepeace, B.L. (2014). Integrated transcriptomic and proteomic analysis of the global response of *Wolbachia* to doxycycline-induced stress. ISME J *8*, 925-37.

S6. Cordero, E.M., Nakayasu, E.S., Gentil, L.G., Yoshida, N., Almeida, I.C. and da Silveira, J.F. (2009). Proteomic analysis of detergent-solubilized membrane proteins from insect-developmental forms of *Trypanosoma cruzi*. J Proteome Res *8*, 3642-3652.

S7. Shevchenko, A., Tomas, H., Havlis, J., Olsen, J.V. and Mann, M. (2006). In-gel digestion for mass spectrometric characterization of proteins and proteomes. Nat Protoc *1*, 2856-2860.

S8. Jones, P.I, Binns, D., Chang, H.Y., Fraser, M., Li, W., McAnulla, C., McWilliam, H., Maslen, J., Mitchell, A., Nuka, G. *et al*. (2014). InterProScan 5: genome-scale protein function classification. Bioinformatics *30*, 1236-1240.

S9. Petersen, T.N., Brunak, S., von Heijne, G. and Nielsen, H. (2011). SignalP 4.0: discriminating signal peptides from transmembrane regions. Nat Methods *8*, 785-786.

S10. Krogh, A., Larsson, B., von Heijne, G. and Sonnhammer, E.L. (2001). Predicting transmembrane protein topology with a hidden Markov model: application to complete genomes. *J Mol Biol 305*, 567-580.

S11. Poisson, G., Chauve, C., Chen, X. and Bergeron, A. (2007). FragAnchor: a large-scale predictor of glycosylphosphatidylinositol anchors in eukaryote protein sequences by qualitative scoring. Genomics Proteomics Bioinformatics *5*, 121-130.

S12. Nawrocki, E.P., Burge, S.W., Bateman, A., Daub, J., Eberhardt, R.Y., Eddy, S.R., Floden, E.W., Gardner, P.P., Jones, T.A., Tate, J. *et al*. (2015). Rfam 12.0: updates to the RNA families database. Nucleic Acids Res *43*, D130-137.

S13. Poliakov, A., Foong, J., Brudno, M. and Dubchak, I. (2014). GenomeVISTA - an integrated software package for whole-genome alignment and visualization. Bioinformatics 30, 2654-2655.

S14. Tamura, K., Stecher, G., Peterson, D., Filipski, A. and Kumar, S. (2013). MEGA6: Molecular Evolutionary Genetics Analysis version 6.0. Mol Biol Evol *30*, 2725-2729.

S15. Faith, D.P. (1992). Conservation evaluation and phylogenetic diversity. Biol Conserv *61*, 1–10.

S16. Minh, B.Q., Klaere, S. and von Haeseler, A. (2009). Taxon Selection under Split Diversity. Syst Biol *58*, 586-594.

S17. Hamby, S.E. and Hirst, J.D. (2008). Prediction of glycosylation sites using random forests. BMC Bioinformatics *9*, 500.

S18. Steentoft, C., Vakhrushev, S.Y., Joshi, H.J., Kong, Y., Vester-Christensen, M.B., Schjoldager, K.T., Lavrsen, K., Dabelsteen, S., Pedersen, N.B., Marcos-Silva, L. *et al*. (2013). Precision mapping of the human O-GalNAc glycoproteome through SimpleCell technology. EMBO J *32*, 1478-1488.

S19. Lobley, A., Sadowski, M.I. and Jones, D.T. (2009). pGenTHREADER and pDomTHREADER: new methods for improved protein fold recognition and superfamily discrimination. Bioinformatics *25*, 1761-1767.

S20. McGuffin, L.J., Bryson, K. and Jones, D.T. (2000). The PSIPRED protein structure prediction server. Bioinformatics *16*, 404-405.
